# Ginsenoside CK and retinol on UVA-induced photoaging exert the synergistic effect through antioxidant and antiapoptotic mechanisms

**DOI:** 10.1038/s41598-025-99304-1

**Published:** 2025-05-13

**Authors:** Jingyin Zhang, Zhuojun Li, Xiaoping Song, Panpan Cai, Qingchao Liu

**Affiliations:** 1 Guangzhou Guangya New Hanfang Cosmetic Technology Co., 18 Tianhui Road, Tianhe District, Guangzhou, 510630 Guangdong China; 2 Guangzhou Guangya New Hanfang Biotechnology Co., 18 Tianhui Road, Tianhe District, Guangzhou, 510630 Guangdong China; 3 Guangzhou Guangya Life Science Research Partnership, Guangzhou, 510630 Guangdong China; 4 Department of Pharmaceutical Engineering, Northwest University, 229 Taibai North Road, Xi’an, 710069 Shaanxi China

**Keywords:** Retinol, Hydroxypinacolone retinoate, Retinol palmitate, Ginsenoside CK, Anti-aging, Biochemistry, Health care

## Abstract

Retinol and retinoids can effectively intervene skin aging process, but usually induce skin intolerance. In this study, we aimed to determine the synergistic anti-aging effects of retinol and two retinol derivatives-hydroxypinacolone retinoate (HPR) and retinol palmitate (VAPA) combined with ginsenoside CK in terms of preventing and treating the UVA radiation-induced skin aging. We found that the combination formulation of retinol and ginsenoside CK alleviated the inhibition of photoaging proliferation of HaCaT cells caused by UVA, and reduced the proportion of senescence. Additionally, the combination of retinol, HPR, VAPA with ginsenoside CK significantly down-regulated the expression of P53 and P21, up-regulated P63 in UVA irradiated cells, and had potential anti-apoptotic activity. Ginsenoside CK intervention also inhibited the degradation of collagen and elastin by reducing the expression of matrix metalloproteinases, and significantly alleviated oxidative stress. Further transcriptomic and molecular docking studies suggested that ginsenoside CK may play an anti-photoaging role by binding to the active pocket of AKR1C1 and AKR1C2 proteins. Zebrafish experiment showed that retinol combined with ginsenoside CK had the effect of reducing skin toxicity. In conclusion, our results show that retinol, HPR and VAPA combined with ginsenoside CK have good anti-aging and irritation-reducing effects in vitro and in vivo.

**Supplementary Information:**

The online version contains supplementary material available at 10.1038/s41598-025-99304-1.

## Introduction

The ultraviolet rays that the sun irradiates to the surface of the earth are mainly composed of UVA and UVB. Among them, UVA, which have a strong penetrating power and can reach the dermis of the skin, destroy elastic fibers and collagen fibers, and cause aging and senescence of the skin^1,2^. Studies have shown that UV radiation increases oxidative stress and induces an inflammatory response in different types of skin cells^3^. UV exposure induces the degradation of extracellular matrix, such as the upregulation of matrix metalloproteinases (MMP1, MMP2, MMP3 and so on), especially the degradation of collagen, which is one of the main causes of skin photoaging^4^.

Retinol, also known as vitamin A, is an important fat-soluble vitamin that protects the skin, maintaining oral health, vision, and immune function, and promoting bone growth and development^5^. Moreover, retinol tends to inhibit the production of metalloproteinase responsible for the extracellular matrix degradation and stimulate the production of collagen I which ensures the maintenance of the skin by the dermis. However, retinol is irritating to the skin and easy to be decomposed by light, heat, oxygen, etc., it is widely used in cosmetics and external medication as a derivative of retinol or retinoids.

Retinoids, as the name suggests, are substances that are similar in structure and function to retinols, which include retinol, retinol palmitate, retinol propionate (RP), retinol retinate, and now the emerging hydroxypinacolone retinoate (HPR)^6^. HPR is a new retinoid that has been shown to be less irritating but highly potent and safe and effective anti-aging and anti-acne ingredient^7^. A study by Bai et al.^8^ showed that HPR supramolecular nanoparticles showed high stability and low irritation. Systemic and comprehensive research on the optimal composition and concentration of retinoids is meaningful to provide powerful anti-aging benefits with minimal irritation. Therefore, there is an urgent need to better understand retinol and its derivatives for developing strategies to combat skin aging^9^.

Ginsenosides, an important class of active components of ginseng, belong to triterpenoid glycosides, which have the functions of enhancing body constitution, regulating nerves and delaying aging^10^. Previous reports have demonstrated that ginsenoside Rc and Rk1 can protect skin against UVB induced photooxidative damage in epidermal keratinocytes^11^. A study by Lim et al.^12^ showed that 20-O-β-D-glucopyranosyl-20(S)-protopanaxadiol, a metabolite of ginsenoside Rb1, enhanced the production of hyaluronic acid through the activation of ERK and Akt mediated by Src tyrosin kinase in human keratinocytes. Moreover, ginsenoside CK has been shown to enhance the transcriptional expression level of filaggrin in HaCaT cells^13,14^. These results provide a reference for exploring the antiphotoaging effect of ginsenosides.

Therefore, the synergistic effect of ginsenoside CK and retinol on UVA-induced photoaging was studied in this paper. We discovered the protective effect of ginsenoside CK on HaCaT keratinocytes after UVA irradiation. The underlying molecular mechanisms of ginsenoside CK by alleviating oxidative stress, inflammatory response, anti-apoptosis, and inhibiting collagen degradation have been verified. Subsequently, transcriptomic techniques were used to predict the potential target proteins of ginsenoside CK anti-aging. Zebrafish experiment in vivo shows that retinol combined with ginsenoside CK has the effect of reducing irritation. These studies suggested that ginsenoside CK combined with retinol could synergistically enhance the anti-photoaging effect and reduce the irritability of retinol, providing a new application direction for the anti-aging application of retinol in cosmetics and external medication.

## Materials and methods

### Reagents

**Fig. 1:**
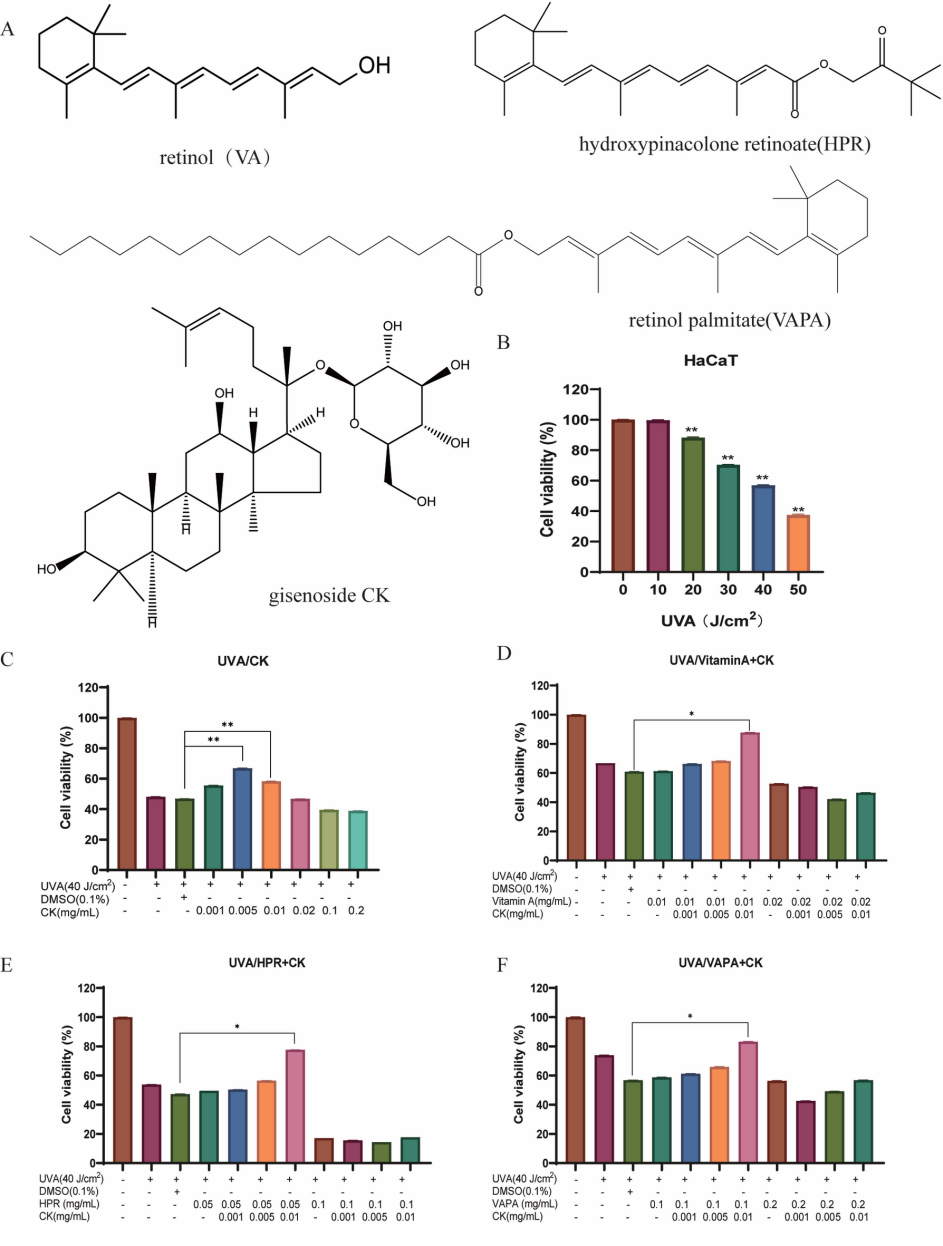
The combination formulation of retinol and CK alleviates the inhibition of photoaging proliferation of HaCaT cells caused by UVA. (**A**) The chemical structures of ginsenoside CK, retinol (Vitamin A, VA), retinol palmitate (VAPA) and hydroxypinacolone retinoate (HPR); (**B**) HaCaT cells were irradiated with different doses of UVA ((0, 10, 20, 30, 40, 50 J/cm^2^), and the effect of UVA irradiation on cell survival rate was detected by MTT method; (**C**) MTT assay were used to investigate the protective effects of ginsenoside CK alone on the proliferation of HaCaT cells during UVA photoaging. (**D**–**F**) MTT assay were used to investigate the protective effects of ginsenoside CK combined with Vitamin A (retinol, 0.01 and 0.02 mg/mL), HPR .(0.05 and 0.1 mg/mL) and retinol palmitate (VAPA, 0.1 and 0.2 mg/mL) on the proliferation of HaCaT cells during UVA photoaging. *P < 0.05; **P < 0.01; ***P < 0.001 versus UVA + DMSO (0.1%) treatment group.

Ginsenoside CK (≥ 50%), retinol (VA, ≥ 50%), retinol palmitate (VAPA, ≥ 99%) and hydroxypinacolone retinoate (HPR, ≥ 99%) were obtained from the Guangzhou Guangya new Hanfang Biotechnology Co., LTD (Guangzhou, China). The chemical structures are shown in Fig. 1A.

### Cell culture and treatment

Human HaCaT cells obtained from the American Type Culture Collection (ATCC, USA) were cultivated in Dulbecco’s modified Eagle’s medium (DMEM) supplemented with 10% fetal bovine serum. In brief, HaCaT cells were cultured in the cell plates, the DMEM medium were removed on the next day, and washed with phosphatebuffered saline (PBS) by twice. Then, HaCaT cells were irradiated by UVA lamps (PL-DY60, Beijing Precise Technology Co., Ltd.) with an emission wavelength peak of 365 nm at different radiation doses (0, 10, 20, 30, 40, 50 J/cm^2^). Irradiation dose (mJ/cm^2^) = UVA irradiation power (mW/cm^2^) × time(s). Subsequently, PBS was removed, and cells were cultured in DMEM with different doses of CK (0.001, 0.005, 0.01, 0.02, 0.1 and 0.2 mg/mL) or Vitamin A (0.01 and 0.02 mg/mL), or HPR (0.05 and 0.1 mg/mL), or VAPA (0.1 and 0.2 mg/mL) for 24 h. The control cells were incubated with DMEM containing 0.1% DMSO. The treated HaCaT cells were then taken out for further assays.

### β-galactosidase staining

In Situ β-galactosidase Staining Kit was purchased from Beyotime Biotechnology (RG0039, Shanghai, China). HaCaT cells were irradiated with 40 J/cm^2^ doses of UVA and treated with different drugs for 24 h. Then, the cell culture medium was sucked, 1 ml β-galactosidase staining fixing solution was added and fixed at room temperature for 10 min. The cell fixative was removed and the cells were washed with PBS 3 times for 3 min each time. PBS was absorbed and 1 mL of dyeing solution was added to each well and incubated at 37 °C for 2 h. Observed and counted under a normal light microscope.

### Western blot analysis

Cells were irradiated with 40 J/cm^2^ doses of UVA and incubated with ginsenoside CK or Vitamin A, HPR, VAPA at the indicated concentrations for 24 h, washed with twice PBS, lysed on ice for 30 min in loading buffer and then boiled for approximately 10 min. The protein concentration was determined with a BCA reagent. Equal amounts of protein were electrophoresed in 6–10% SDS-PAGE gels, transferred to PVDF membranes (Pall), probed with antibodies and detected by chemiluminescence (Pierce). Image J software from the National Institutes of Health (Wayne Rasband, USA) was utilized to quantify the bands’ intensity.

Antibodies against PARP1 (#13371-1-AP), Caspase 8(#13423-1-AP), Caspase9(#10380-1-AP), P53(#60283-2-Ig), P21(#10355-1-AP), P63(#12143-1-AP), Elastin(#15257-1-AP), Collagen-I(#14695-1-AP), MMP2(#10373-2-AP), MMP3(#17873-1-AP) and GAPDH (#10494-1-AP) were obtained from Proteintech (Wuhan, China).

### Cell reactive oxygen species (ROS) detection

The ROS detection kits were purchased from Beyotime Biotechnology (Shanghai, China). The blank group, the positive control group, and five experimental groups: control group, UVA group, CK group (0.01 mg/mL), VA/HPR/VAPA alone group (0.01/0.05/0.1 mg/mL), CK + VA/HPR/VAPA, and the cells were inoculated in six-well plates at a density of 5 × 10^5^ cells/well, incubating for 24 h. After washing twice with PBS, cells were irradiated with 40 J/cm^2^ doses of UVA and treated with certain drugs for 24 h. Rosup was added to the positive control group 30 min in advance to stimulate cells. Subsequently, all groups were washed, digested, and centrifuged to collect cells. Repeat PBS resuspension twice to avoid the influence of serum in the culture medium on probe binding. The probe was diluted by serum-free medium at a ratio of 1:1000 (the blank group without probe). Cells were incubated with probes for 20 min and inverted every 5 min to ensure completely contact. After incubation, centrifuged the cells and washed twice with PBS and three times with serum-free culture medium to remove the unadsorbed probes. Finally, the cells were resuspended with 1 mL PBS and detected by Flow cytometry.

### Determination of the antioxidant profiles of HaCaT cells

Cells proteins were extracted, and the levels of MDA, SOD, CAT were examined by commercial detection kits (Solarbio, Beijing, China) as described above according to the manufacturer’s protocols. The protein extraction process of these three kits was as follows: The cells were inoculated in a culture dish and incubated for 24 h. After irradiating with 40 J/cm^2^ doses of UVA, the cells were treated with different drugs for another 24 h. The cells were collected in centrifuge tubes, and the supernatant was discarded after centrifugation. According to the cell counting results, added 2 mL extraction solution to resuspend the cells followed by sonicating the cells (200W, ultrasound for 3 s, interval of 10 s, repeated 30 times) under ice bath condition. The samples were centrifuged at 8000 g, 4 °C for 10 min. The supernatant was retained and stored at − 20 °C.

### RNA-seq assay

Cells were inoculated in a six-well plate at a density of 2 × 10^5^ cells per well, and three experimental groups were set up: 0 UVA, UVA, and UVA + CK 0.005. After 24 h, the cells were irradiated, treated with drugs and continued to be incubated. Using TRIzol lysis method to extract cell RNA. Samples were sent to Shenzhen BGI Co., Ltd. for testing and analysis using the DNBSEQ platform.

BGI multi-omics system was used for data analysis. In short, RNA sequencing data was imported into the system, the filtering software SOAPnuke was used to preprocess the data, including standardization and missing value processing. Then, the quantitative analysis of genes, various analyses based on gene expression level, including PCA, correlation, differential gene (DEG) screening, etc. Moreover, the DEGs among the selected samples were further mined and analyzed, including VENN map, cluster heat map, KEGG signal pathway enrichment analysis map and GO analysis map.

### Molecular docking

The crystal structures of AKR1C1 (PDB code: 1MRQ), AKR1C2 (PDB code: 1IHI), HSPA6 (PDB code: 3FE1), and SCD (PDB code: 4ZYO) were acquired from the Protein Data Bank. ChemDraw was utilized to draw the three-dimensional structure of CK. AKR1C1, AKR1C2, HSPA6 and SCD and CK was docked using AutoDock Vina 4.2. The protein and ligand were obtained by AutoDock Tools. The docking results were examined with the PyMOL molecular graphics system.

### In vivo zebrafish experiment

Wild-type (AB-strain) zebrafish were obtained from the Beijing Hongdagaofeng Aquarium Department for this portion of the study. Adult zebrafish were acclimatized in a flow-through feeding equipment (Esen Corp., China) under a 14-h light/10-h dark cycle at a temperature of 27 ± 1 °C and a pH of 7.5 ± 0.5 for a period of 2 weeks. The zebrafish were fed live brine shrimp twice daily. Embryos were collected from spawning adult zebrafish with a sex ratio of 1:2 (female to male) in spawning boxes (Esen Corp, China). Spawning was induced in the morning by activating the light. Within 30 min post-spawning, embryos were collected and rinsed with reconstituted water. Fertilized and normally developed embryos at approximately 3 h post-fertilization (hpf) were staged under a dissecting microscope and subsequently exposed to a range of concentrations of the test substances in aqueous solution. During the exposure test, the water conductivity was maintained at 600 ± 100 µS/cm, and sufficient oxygen levels (> 80%) were ensured. Zebrafish embryos at 3 hpf were subjected to exposure across seven main groups for each test substance: (1) Retinol (VA). (2) Ginsenoside CK. (3) Combination of Retinol and CK. (4) Hydroxypinacolone Retinoate (HPR). (5) Combination of HPR and CK. (6) Vitamin A palmitate (VAPA). (7) Combination of VAPA and CK. The acute toxicity of the test substances was evaluated by monitoring embryo mortality. Zebrafish embryos were observed every 24 h, with the number of deaths recorded. The criteria for determining embryo death included the presence of a milky coagulum or developmental stasis.

### Statistical analysis

All results were independently replicated a minimum of three times. The error bars on the graphs display the standard deviation (SD). GraphPad Prism software version 9 was used for statistical analysis. The results were analyzed using a t-test and a one-way ANOVA for multiple groups, where *P* < 0.05 was considered statistically significant.

## Results

### The combination formulation of retinol and CK alleviates the inhibition of photoaging proliferation of HaCaT cells caused by UVA

In order to establish the UVA photoaging model of HaCaT cells, the HaCaT cells were irradiated with different doses of UVA ((0, 10, 20, 30, 40, 50 J/cm^2^), and the effect of UVA irradiation on cell survival rate was detected by MTT method. Effects of ginsenoside CK, retinol (Vitamin A, VA), hydroxypinacolone retinoate (HPR) and retinol palmitate (VAPA) on cytotoxicity of HaCaT cells were determined using MTT assay (Fig. S1). As shown in Fig. 1B, HaCaT cells exposed to UVA had a reduced survival rate compared to the untreated group. With the increase of UVA irradiation dose, the viability of HaCaT cells decreased. A radiation dose that inhibited HaCaT cell survival activity by 50%, i.e. 40 J/cm^2^ irradiation, was selected for follow-up experiments.

Next, we investigated the protective effects of ginsenoside CK alone and its combination with retinol on the proliferation of HaCaT cells during UVA photoaging. First of all, compared with the group without UVA radiation, the cell survival rate after UVA radiation was only about 50%, indicating that the photoaging cell model was successfully constructed (Fig. 1C). Then, compared with UVA group, ginsenoside CK alone at low concentrations of 0.005 mg/mL and 0.01 mg/mL could significantly promote the proliferation of HaCaT cells after UVA radiation. Therefore, we selected ginsenoside CK concentrations of 0.001, 0.005 and 0.01 mg/mL in the combined administration regimen. As shown in Fig. 1D–F, Vitamin A(retinol, 0.01 mg/mL), HPR(0.05 mg/mL) and retinol palmitate (VAPA, 0.1 mg/mL) alone could not increase the proliferation activity of HaCaT cells after UVA radiation, while the combination with CK of different concentration gradients could promote the proliferation activity of HACAT cells to varying degrees. *P < 0.05, **P < 0.01, versus UVA + DMSO (0.1%). Among them, Vitamin A (0.01 mg/mL) combined with CK (0.01 mg/mL), HPR (0.05 mg/mL) combined with CK (0.01 mg/mL), VAPA (0.1 mg/mL) combined with CK (0.01 mg/mL) had the most significant effect on the proliferation of photoaged HaCaT cells.

### The combination formulation of retinol and CK alleviates UVA-induced senescence of HaCaT cells

**Fig. 2:**
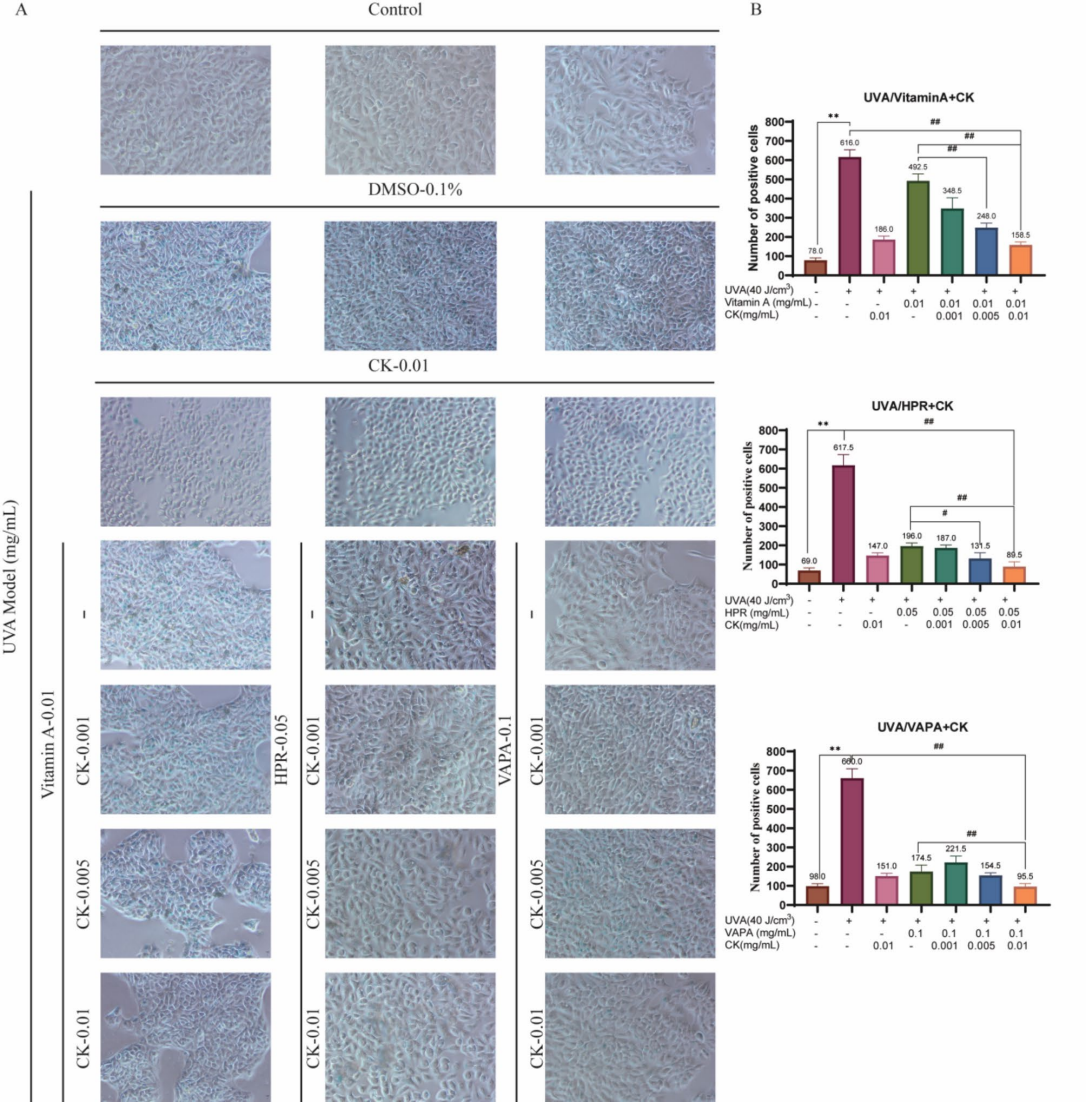
The combination formulation of retinol and CK alleviates UVA-induced senescence of HaCaT cells. (**A**) The proportion of senescent cells after ginsenoside CK alone or combined with VA, HPR or VAPA was observed by β-galactosidase staining. ImageJ software was used to count the number of cells with positive staining, and the quantitative statistical graph was shown in (**B**). **P < 0.01 versus control treatment group. ^#^*P* < 0.05, ^##^*P* < 0.01 versus UVA treatment group.

To detect the effect of the combination of retinol and CK on the senescence level of UVA-induced aging HaCaT cells, β-galactosidase staining was used to observe the proportion of senescent cells. As shown in Fig. 2A, β-galactosidase staining was significantly deepened in the UVA irradiation group compared with the control group. However, compared with UVA irradiation group, Vitamin A, HPR and VAPA combined with ginsenoside CK significantly reduced β-galactosylase staining levels, suggesting that Vitamin A, HPR and VAPA combined with CK could alleviate UVA-induced aging of HaCaT cells. ImageJ software was used to count the number of cells with positive staining, and the quantitative statistical graph was shown in Fig. 2B.

### The combination of retinol and CK alleviates the expression levels of senescence-related proteins

**Fig. 3:**
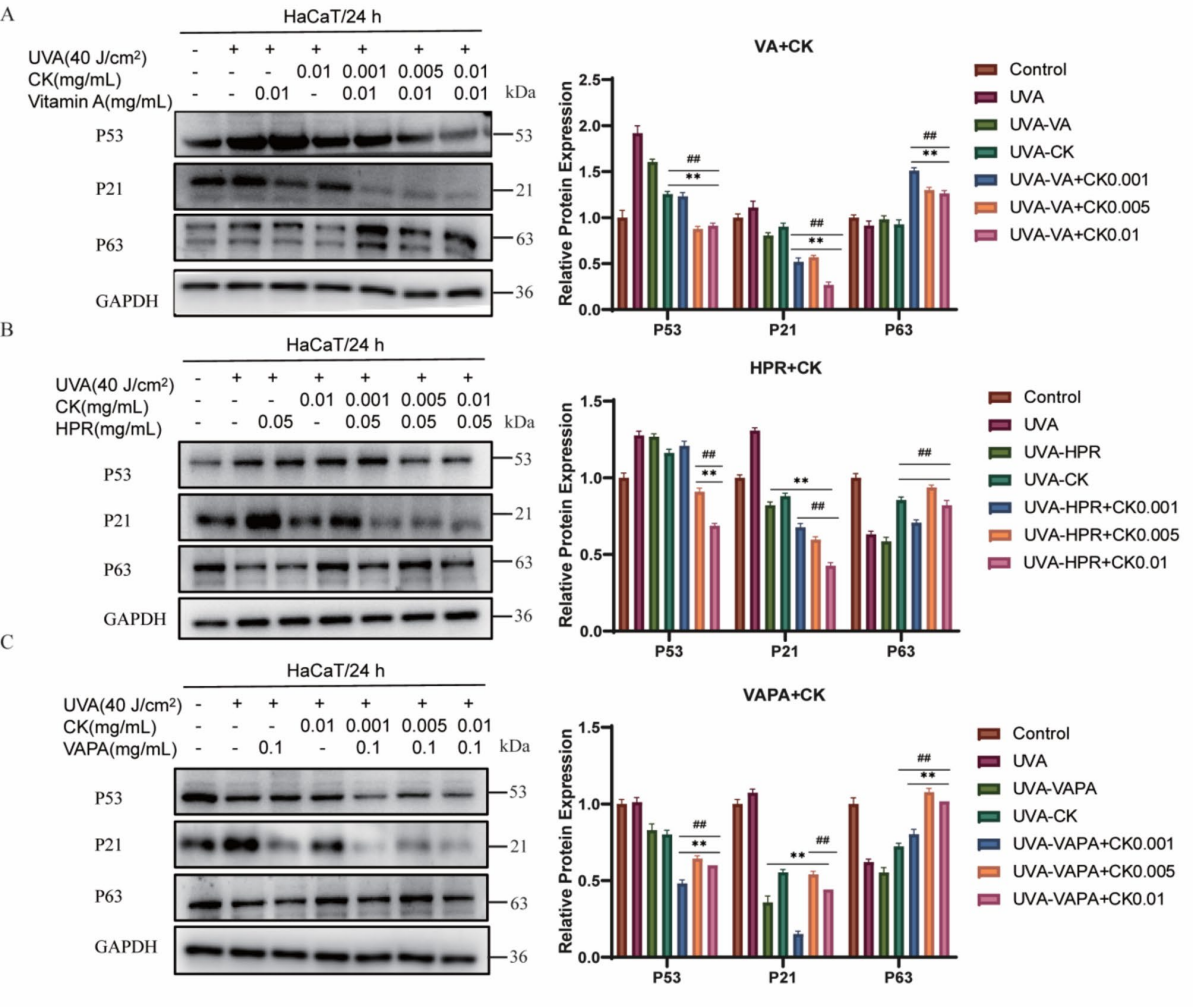
The combination of retinol and CK alleviates the expression levels of senescence-related proteins. (**A**) The effects of the combination of CK and VA on the expression levels of age-related proteins were detected by Western Blot. (**B**)The effects of the combination of CK and HPR on the expression levels of age-related proteins were detected by Western Blot. (**C**)The effects of the combination of CK and VAPA on the expression levels of age-related proteins were detected by Western Blot. Protein expression levels of P53, P21 and P63 were determined using Western blot analysis in HaCaT cells treated with CK (0.001, 0.005, 0.01 mg/mL) with or without VA (0.01 mg/mL), HPR (0.05 mg/mL) or VAPA (0.1 mg/mL) for 24 h. GAPDH was used as a control protein. Quantification charts were listed on the right. **P < 0.01 versus UVA treatment group. ^#^*P* < 0.05, ^##^*P* < 0.01 versus VA/HPR/VAPA alone treatment group.

Cell senescence is a state of irreversible cell cycle arrest that has been suggested to regulate the process of tumor suppression and aging. The effects of the combination of retinol and CK on the expression levels of age-related proteins were detected by Western Blot. As shown in Fig. 3A–C, UVA significantly increased the expression of HaCaT cell senescence related proteins P53 and P21. While, the combination of retinol and HPR, VAPA with CK can significantly down-regulate the expression of P53 and P21 in UVA irradiated cells. In addition, it has recently been found that the loss of the p53-associated protein P63 induced cellular senescence and led to features of accelerated aging^15^. Results showed that UVA irradiation caused the down-regulation of P63 protein expression in HaCaT cells, and the combination of retinol and retinoid with CK could significantly up-regulate the expression of P63 in UVA irradiated cells. Quantitative analysis was shown on the right.

### The combination of retinol and CK decreased UVA-induced apoptosis of HaCaT cells

**Fig. 4:**
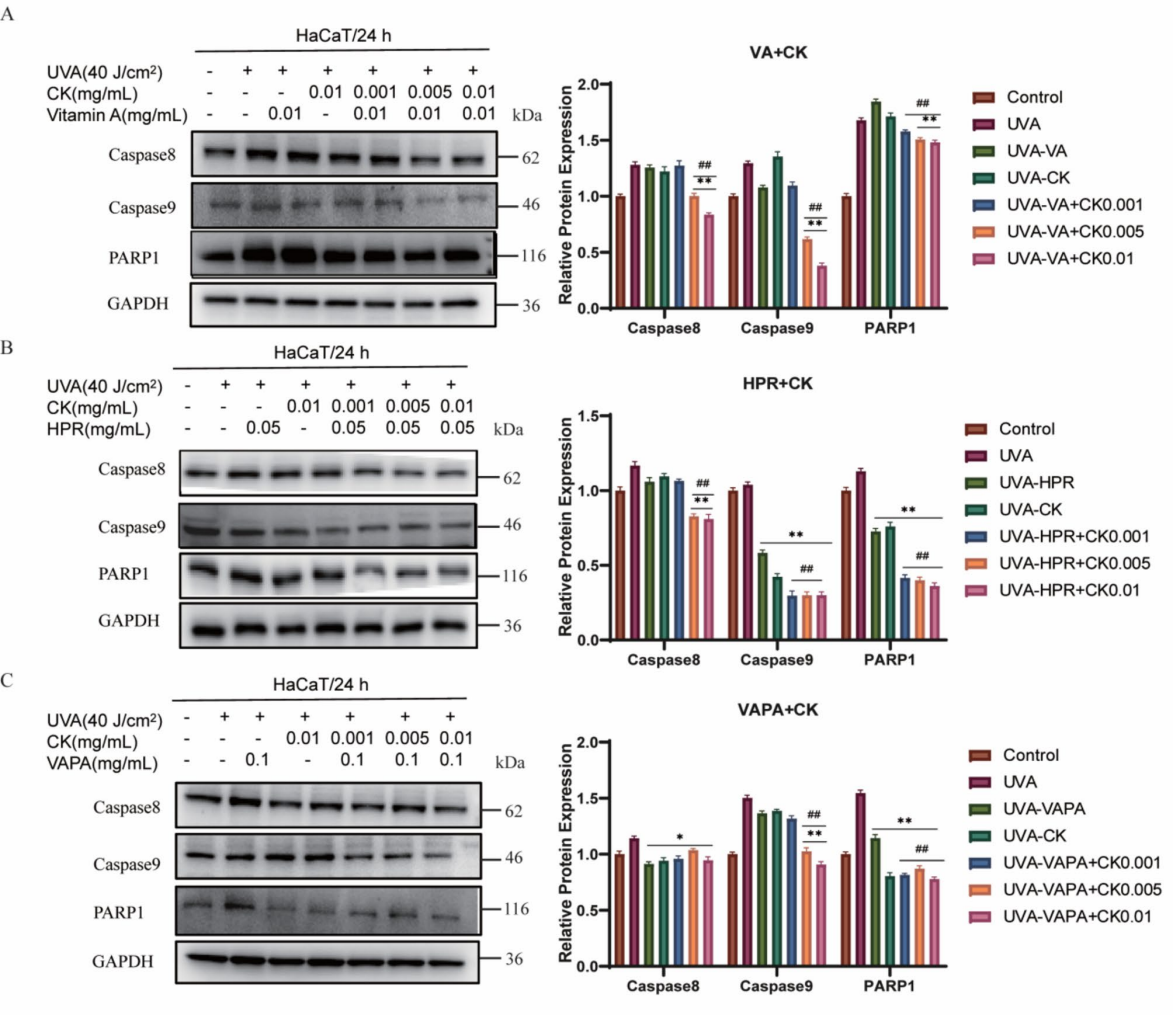
The combination of retinol and CK decreased UVA-induced apoptosis of HaCaT cells. (**A**) The effects of the combination of CK and VA on the expression levels of apoptosis-related proteins were detected by Western Blot. (**B**) The effects of the combination of CK and HPR on the expression levels of apoptosis-related proteins were detected by Western Blot. (**C**) The effects of the combination of CK and VAPA on the expression levels of apoptosis-related proteins were detected by Western Blot. Protein expression levels of Caspase 8, Caspase 9 and PARP1 were determined using Western blot analysis in HaCaT cells treated with CK (0.001, 0.005, 0.01 mg/mL) with or without VA (0.01 mg/mL), HPR (0.05 mg/mL) or VAPA (0.1 mg/mL) for 24 h. GAPDH was used as a control protein. Quantification charts were listed on the right. **P < 0.01 vs UVA treatment group. ^#^*P* < 0.05, ^##^*P* < 0.01 versus VA/HPR/VAPA alone treatment group.

The results of western blot on the apoptosis pathway (Fig. 4) showed that the cells induced by UVA radiation overexpressed caspase 8, caspase 9 and PARP. However, this situation was reversed under different concentrations of CK combined with retinol. Combined treatment of retinol (VA), HPR, VAPA and CK dose-dependently down-regulated the protein expression of caspase 8, caspase 9 and PARP. Quantitative analysis was shown on the right. The regulation of apoptotic signaling pathway related proteins indicated that the combination of retinol and CK had potential anti-apoptotic activity in photoaging cells.

### The combination formulation of retinol and CK promotes the increase of collagen and elastin in photoaged cells

**Fig. 5:**
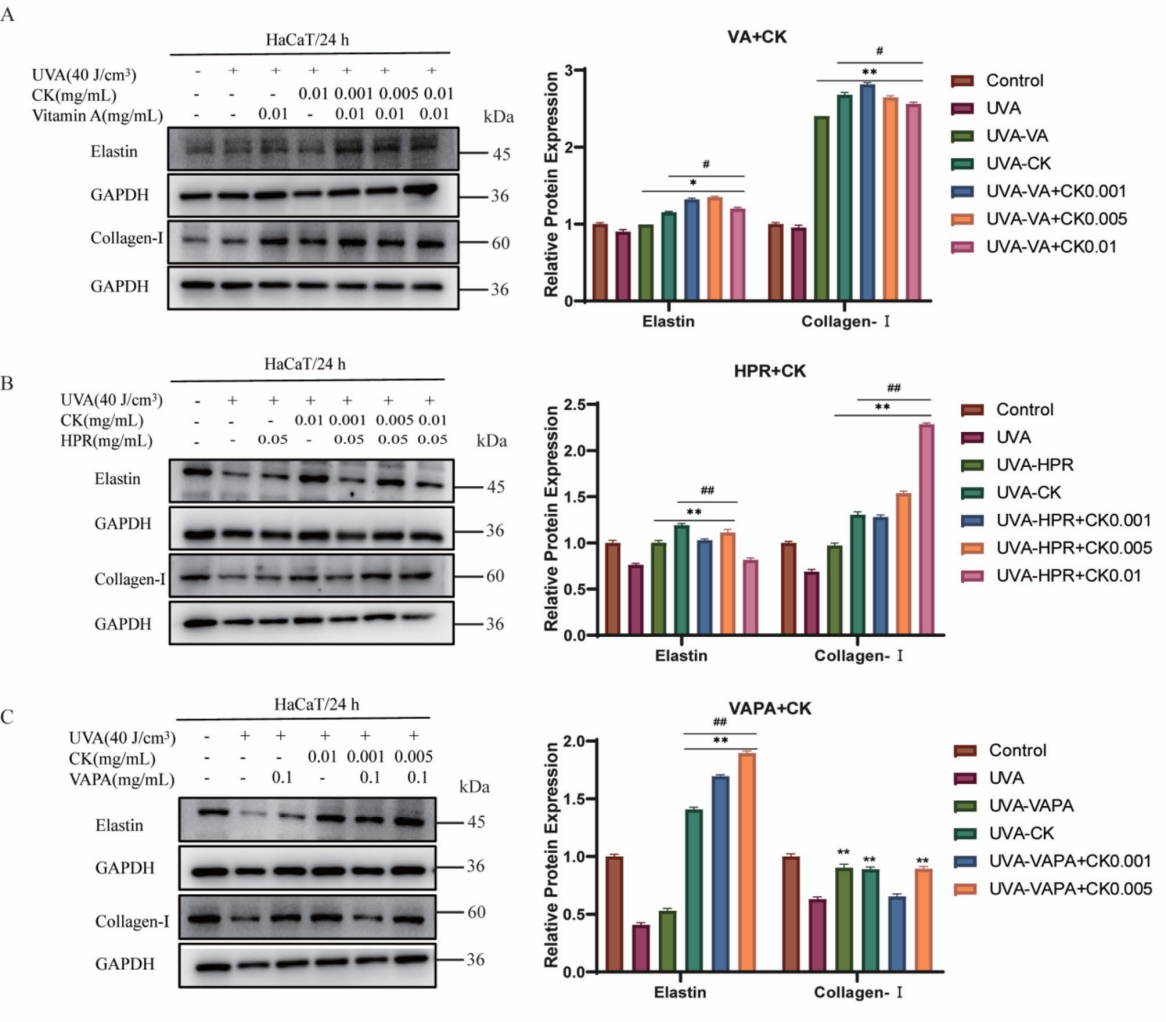
The combination formulation of retinol and CK promotes the increase of collagen and elastin in photoaged cells. (**A**–**C**) Western Blot assay was used to investigate the anti-aging effect of the combination of CK and retinol by detecting the expression levels of type I collagen and elastin. Protein expression levels of Collagen-I and Elastin were determined using Western blot analysis in HaCaT cells treated with CK (0.001, 0.005, 0.01 mg/mL) with or without VA (**A**), HPR (**B**) or VAPA (**C**) for 24 h. GAPDH was used as a control protein. Quantification charts were listed on the right. **P < 0.01 vs UVA treatment group. ^#^*P* < 0.05, ^##^*P* < 0.01 versus VA/HPR/VAPA alone treatment group.

Collagen is essential components for maintaining normal body activities, ae well as substance that keeps the body young and prevents aging^16^. Elastin is the main component of elastic fibers and plays an important role in skin elasticity^17^. Therefore, we investigated the anti-aging effect of the combination of CK and retinol by detecting the expression levels of type I collagen and elastin. As indicated in Fig. 5A–C, compared to the untreated cells, UVA irradiation induced the down-regulation of Collagen I and Elastin expression in HaCaT cells. Ginsenoside CK combined with retinol increased the expression of Collagen I and Elastin in a concentration gradient-dependent manner. ImageJ software was used for quantitative analysis, and the results were shown on the right.

Matrix metalloproteinases (MMPS) are a kind of proteinases that are highly conformed in nature, which lead to excessive degradation of collagen and elastin in the dermis that support the skin structure, resulting in aging symptoms such as wrinkles and decreased elasticity^18^. Therefore, the effects of ginsenoside CK combined with retinol and retinoid on matrix metalloproteinases in photoaged cells were detected by Western Blot. As shown in Supplementary Fig. 2A–C, compared with the control group, UVA irradiation had no significant effect on or slightly increased the expression of MMP2 in HaCaT cells, but combined treatment with ginsenoside CK could reduce MMP2 expression in a concentration gradient-dependent manner. Similarly, UVA irradiation led to the upregulation of MMP3 expression, whereas combined treatment with ginsenoside CK reduced MMP3 expression in a concentration gradient-dependent manner. ImageJ software was used for quantitative analysis, and the results were shown on the right.

### The combination formulation of retinol and CK alleviates the oxidative stress level of photoaged cells

**Fig. 6:**
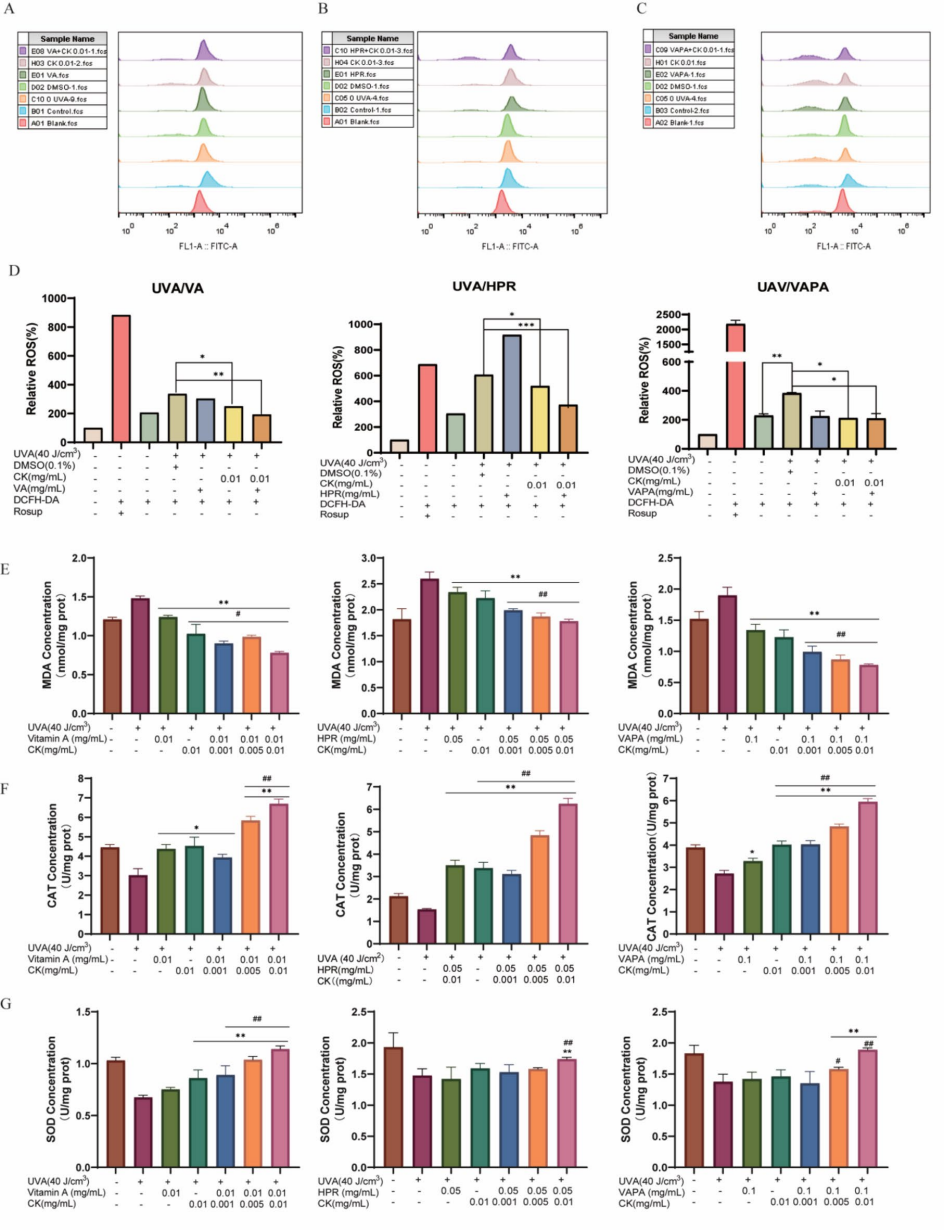
The combination formulation of retinol and CK alleviates the oxidative stress level of photoaged cells. (**A**–**C**) Flow cytometry was used to detect the effects of CK and retinol on ROS. HaCaT cells were treated with CK (0.001, 0.005, 0.01 mg/mL) with or without VA (0.01 mg/mL), HPR (0.05 mg/mL) or VAPA (0.1 mg/mL) for 24 h. And then fluorescent probe DCFH-DA was used to detect ROS. Quantification charts were listed on (**D**). (**E**–**G**) UVA-irradiated human HaCaT cells were treated with CK with and without VA, HPR or VAPA. The quantitative analysis of MDA (**E**), CAT (**F**) and SOD (**G**). **P < 0.01 versus UVA treatment group. ^#^*P* < 0.05, ^##^*P* < 0.01 versus VA/HPR/VAPA alone treatment group.

Reactive oxygen species (ROS) could damage and harden collagen in the dermis. Therefore, the effects of the combination of retinol and retinoid with CK on ROS were examined. As shown in Fig. 6, compared with Rosup probe blank control, ROS increased significantly after UVA radiation, indicating successful modeling. Compared with UVA radiation group (Fig. 6A), ROS was not significantly decreased in VA (0.01 mg/mL) group alone, however, ROS was significantly decreased after ginsenoside CK (0.01 mg/mL) treatment, and ROS was significantly decreased when the two were combined. The results were similar in the combination group of HPR (Fig. 6B) or VAPA (Fig. 6C) and ginsenoside CK. There was no significant decrease in ROS in HPR (0.05 mg/mL) or VAPA (0.1 mg/mL) alone group, but ROS was significantly decreased after combined treatment with ginsenoside CK (0.01 mg/mL). ImageJ software was used for quantitative analysis, and the results were shown in the Fig. 6D.

Oxidative stress and inflammatory factors play a major role in skin aging. As shown in Fig. 6E, compared to the control group, the contents of MDA were markedly increased in UVA-irradiated HaCaT cells, while combined with application of CK effectively recovered the contents of MDA. The levels of SOD (Fig. 6F) and CAT (Fig. 6G), in HaCaT cells were remarkable downregulated by UVA irradiation compared with the control group, whereas CK combined treatment prevented the UVA-induced reductions in SOD and CAT production. These data indicated that CK treatment enhanced the antioxidant ability of UVA-irradiated HaCaT cells.

### The anti-photoaging target of ginsenoside CK by transcriptomics

**Fig. 7:**
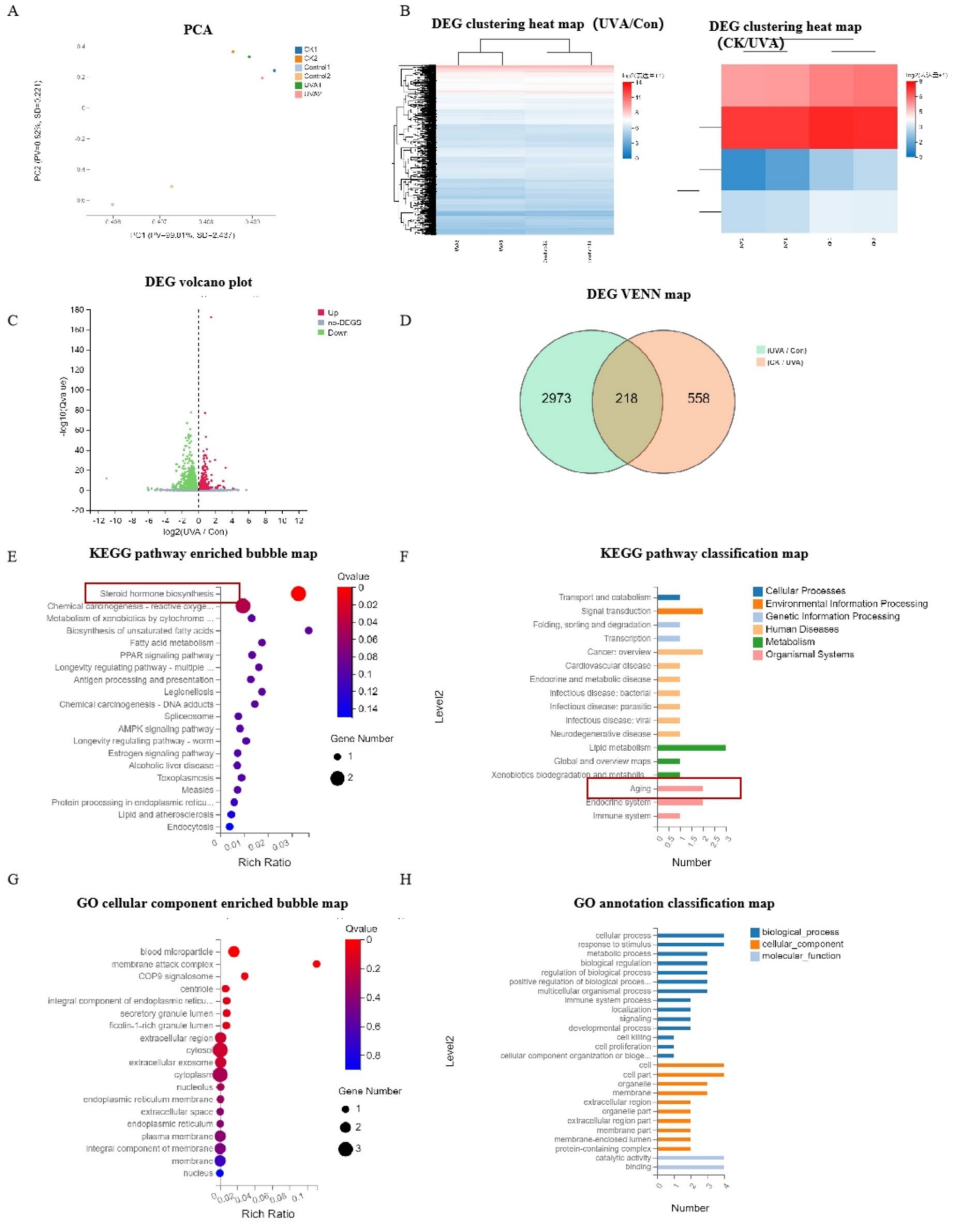
Analysis of transcriptome results of ginsenoside CK. HaCaT cells were irradiated with UVA (40 J/cm^2^) and treated with CK for 24 h. (**A**) PCA analysis of control, UVA and CK group. (**B**) Differential gene (DEG) enrichment analysis. (**C**) Volcano map of DEGs (Fold change ≥ 2 and *P* < 0.05). (**D**) DEG VENN map. (**E**) KEGG pathway enriched bubble map. (**F**) KEGG pathway classification map. (**G**) GO enrichment analysis of the DEGs. (**H**) GO annotation classification map of the DEGs.

The mechanism of ginsenoside CK anti-photoaging was investigated by transcriptomics. The principal component analysis (PCA) results revealed that the control and CK groups were clustered and none of the three samples were outliers (Fig. 7A). The UVA treatment group had 2692 DEGs compared to the control group, with 1153 up-regulated and 1539 down-regulated DEGs (Fig. 7B,C). DEG VENN diagram showed that there were 218 intersectional transcripts between CK treatment group and UVA radiation group (Fig. 7D). Of these, four intersection genes were AKR1C1, AKR1C2, HSPA6 and SCD. KEGG pathway^19,20^ enriched bubble map showed that DEG were mainly concentrated in steroid hormone biosynthesis, reactive oxygen, fatty acid metabolism etc., and were related to signal transduction, tumor, cardiovascular disease, aging and other diseases. (Fig. 7E,F). The GO analysis results suggested that ginsenoside CK could be essential in anti-photoaging by influencing these biological processes (Fig. 7G,H).

### Molecular docking ginsenoside CK and AKR1C1, AKR1C2, HSPA6 and SCD protein

**Fig. 8:**
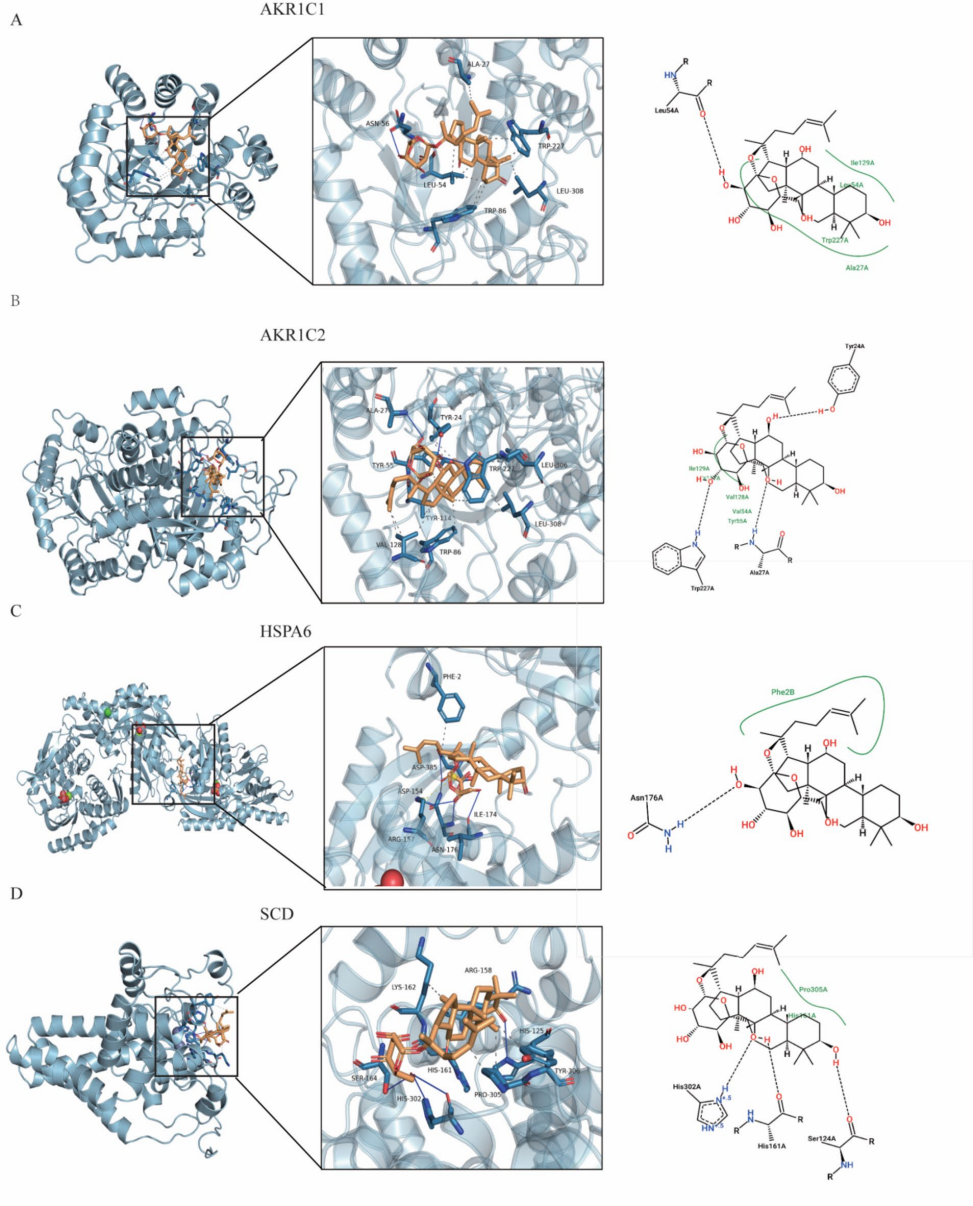
Molecular docking ginsenoside CK and AKR1C1, AKR1C2, HSPA6 and SCD protein. (**A**) Molecular docking simulation results for CK with AKR1C1 protein. (**B**) Molecular docking simulation results for CK with AKR1C2 protein. (**C**) Molecular docking simulation results for CK with AKR1C1 protein. (**D**) Molecular docking simulation results for CK with SCD protein. The residues are shown in green sticks, and the ligand is show in orange sticks.

To investigate the possible mechanism of ginsenoside CK binding with AKR1C1, AKR1C2, HSPA6 and SCD separately, molecular docking study was conducted. The docking data suggested that CK interacted with the active pocket of the receptor protein AKR1C1 through residues Ala27, Asn56, Leu54, Trp227, Trp 86 and Leu308 via the hydrogen bond, respectively (Fig. 8A). Additionally, CK interacted with the active pocket of the receptor protein AKR1C2 through residues Ala27, Tp227 and Tyr24 by the hydrogen bond and hydrophobic interactions (Fig. 8B). Moreover, CK bound to the active pocket of the receptor HSPA6 and SCD1, and involved the hydrogen bond (interaction with Asn176, Phe2, and His302, Ser124, respectively) (Fig. 8C and D).

The binding free energies of CK with AKR1C1, AKR1C2, HSPA6 and SCD were − 6.9, − 9.2, − 8.7, and − 7.7 kcal/mol, respectively. Marín et al.^21^ illustrated that ultraviolet light increased the expression of AKR1C1 and AKR1C2 in keratinocytes, human skin explants, and pig skin. Martin et al.^22^ also found that overexpression of AKR1C2 increased the frequency of postaging tumorigenesis (PSNE). According to the transcriptomic results, the expression of AKR1C1 and AKR1C2 were increased after UVA irradiation and decreased after CK treatment. Taken together, the data suggested that CK might occupy the binding sites of AKR1C1 and AKR1C2 and thus play an anti-aging role in skin.

### Ginsenoside CK can reduce the toxicity of retinol in vivo zebrafish

**Fig. 9:**
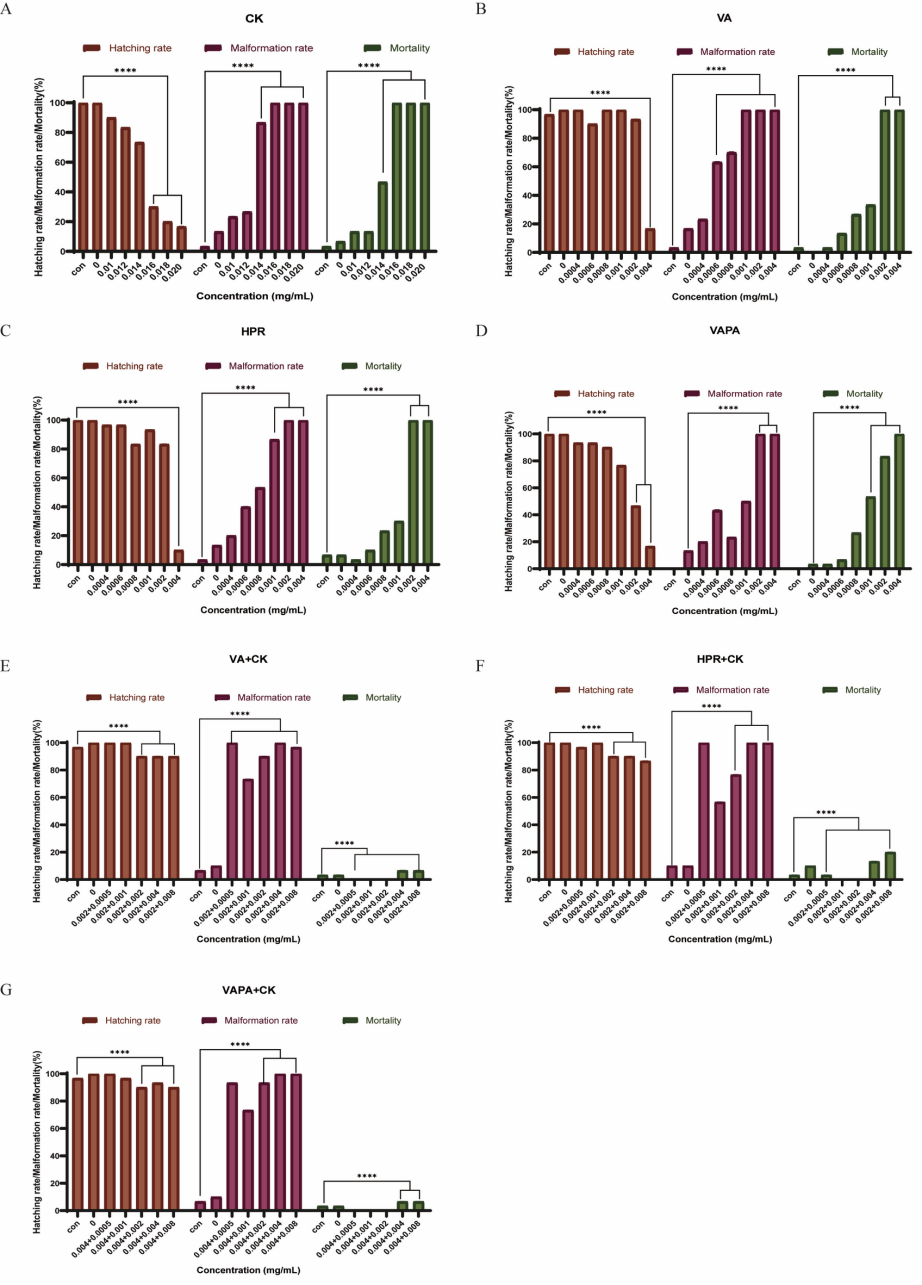
Ginsenoside CK can reduce the toxicity of retinol in vivo zebrafish. (**A**) Effects of CK exposure alone (0, 0.01, 0.012, 0.014, 0.016, 0.018, 0.020 mg/mL) for 96 h on hatchability, malformation rate and mortality of zebrafish embryos. (**B**–**D**) Effects of VA, HPR, or VAPA exposure alone (0, 0.0004, 0.0006, 0.0008, 0.001, 0.002, 0.004 mg/mL) for 96 h on hatchability, malformation rate and mortality of zebrafish embryos. (**E**–**G**) Effects of CK (0, 0.0005, 0.001, 0.002, 0.004, 0.008 mg/mL) combined with VA (0.002 mg/mL), HPR (0.002 mg/mL) or VAPA (0.004 mg/mL) exposure for 96 h on incubation rate, malformation rate and mortality of zebrafish embryos. The results were expressed by means ± standard error (mean ± SEM). Significance analysis was conducted using One-way ANOVA, and *P* < 0.05 was considered as significant difference. ****P < 0.0001.

Finally, zebrafish experiment in vivo was used to detect the effect of ginsenoside CK on reducing toxicity and irritation of retinol. Effects of CK combined with retinol exposure on hatchability, malformation rate and mortality of zebrafish embryos were detected. The results are shown in Fig. 9 below. Figure 9A–D showed the effects of CK, retinol, HPR, or VAPA alone exposure for 96 h on zebrafish embryos. Ginsenoside CK had significant effects on hatching rate, teratogenic rate and mortality of zebrafish embryos at higher doses (0.016, 0.018, 0.020 mg/mL). Retinol, HPR and VAPA had significant effects on hatching rate, teratogenic rate hatching rate, and mortality of zebrafish embryos at higher doses (0.002 and 0.004 mg/mL). Next, CK combined with retinol was applied to zebrafish embryos to observe the effects on zebrafish embryos. Figure 9E showed that the hatchability of zebrafish embryos increased from 20 to 100% after retinol (0.002 mg/mL) combined with CK (0.008 mg/mL) compared with retinol alone, and the mortality rate from 100% to less than 10%. Similarly, the combination of CK with HPR (Fig. 9F) or VAPA (Fig. 9G) can also increase the hatching rate of zebrafish embryos and reduce the teratogenic rate and mortality. These results indicated that the combination with ginsenoside CK reduced the toxicity and irritation of retinol and its derivatives in vivo.

## Discussion

UVA can directly cause photoaging of skin dermis, resulting in damage of fibroblasts, decreased collagen synthesis ability, and destruction of tissue structure^23,24^. Therefore, protecting skin cells from UVA damage is a necessary condition for reducing skin photoaging, and many attempts have been made to explore substantial and effective anti-photoaging interventions^24^.Retinol have been shown to prevent photoaging, but have certain skin irritations. Therefore, there is an urgent need for effective and safe anti-photoaging drug therapy.

Cell photoaging is characterized by cell cycle arrest, up-regulated expression of cell cycle inhibitors P53 and P21, inactivation of apoptosis-related proteins, and cell stasis^25^. The study found that the loss of the P63 gene, a homologous tumor suppressor gene of the P53 gene, accelerated aging in mice^15,26^. Our study found that UVA radiation led to the decrease of p63, and the combination with CK promoted the increase of P63 to a certain extent.

UV-induced skin aging involves increased degradation of ECM by various cell types, including dermal fibroblasts and keratinocytes^27^. A study by Lee et al.^28^ showed that ginsenoside CK exerted the skin protective effect by inhibiting the transcriptional expression of MMP1. Our results show that UVA irradiation leads to degradation of collagen and elastin, while also increasing MMPs. Combined with CK can reverse the degradation of collagen and elastin, and promote the degradation of MMPs.

Excessive UVA irradiation of skin cells can lead to a large accumulation of intracellular ROS, destroy the balance between skin oxidation and antioxidant defense systems, thereby reducing the activity of antioxidant enzymes and inducing oxidative stress response^29,30^. Consistent with this finding, our study showed that combined administration with CK significantly inhibited the overproduction of ROS in HaCaT keratinocytes during UV irradiation. In the case of oxidative stress, intracellular lipids are oxidized and a large amount of MDA is produced, resulting in an increase in the degree of oxidative stress within the cell^31^. Our study found that MDA content of HaCaT cells was significantly increased after uva irradiation, and MDA content could be effectively restored by combining with CK. In addition, antioxidant enzymes (such as CAT and SOD) can protect cells from oxidative damage by catalyzing the breakdown of hydrogen peroxide and reducing the number of superoxide anion radicals^32^. Our results showed that, compared with the UVA irradiation group, the combined application of CK significantly reduced the activities of SOD and CAT, indicating that CK had a protective effect on the skin oxidative damage caused by UVA.

Mechanically, in order to explore the molecular target of CK against skin photoaging, transcriptomic sequencing technology was conducted. The results of KEGG pathway enrichment and GO analysis showed that DEG was mainly concentrated in steroid hormone biosynthesis, reactive oxygen species, fatty acid metabolism, and was associated with signal transduction, tumor, cardiovascular disease, aging and other diseases. Furthermore, RNA-seq differentially expressed genes were enriched in AKR1C1, AKR1C2, HSPA6 and SCD proteins. Molecular docking results showed that CK could bind to AKR1C1, AKR1C2, HSPA6 and SCD proteins to a certain extent. Among them, the binding free energy with AKR1C1 protein was the lowest. Aldoketo reductase family member C1 and C2 (AKR1C1, AKR1C2), were reported to be dysregulated in a variety of tumors, such as lung cancer^33^, cervical cancer^34^, and gastric cancer^35^. Recent studies have found that the expression of HSPA6 mRNA involved in oxidative stress response is increased in healthy elderly people, suggesting that HSPA6 may be related to cell aging^36,37^. Stearoyl CoA desaturase-1 (SCD), a key regulator of fatty acid metabolic pathways, plays multiple roles in cancer, metabolism, and iron death^38^. It was found that the expression of the target gene SCD in the epidermis of human skin was significantly reduced after photoaging or acute UV exposure^39^. Combined with transcriptomics and molecular docking results, expressions of these four proteins were increased after UVA irradiation of CK. Further experiments are needed to further prove the target of CK anti-photoaging.

In conclusion, we found that the synergistic effect of Ginsenoside CK and retinol on reduced photoaging of HaCaT keratinocytes under UVA irradiation was achieved by reversing oxidative stress, apoptosis, and collagen degradation.

## Electronic supplementary material

Below is the link to the electronic supplementary material.

Supplementary Material 1

Supplementary Material 2

## Data Availability

The datasets generated and/or analysed during the current study are available in the NCBI repository, https://www.ncbi.nlm.nih.gov/biosample/. BioSample accessions: SAMN45531777, SAMN4531778, SAM45531779, SAMN45531780, SAMN45531781, SAMN45531782. The datasets generated and/or analysed during the current study will not be publicly available until 2026–10-31, but are available from the corresponding author on reasonable request.
