# Supplementary material for: Ginsenoside CK and retinol on UVA-induced photoaging exert the synergistic effect through antioxidant and antiapoptotic mechanisms
Source: Sci Rep. 2025 May 13;15:16664. doi: 10.1038/s41598-025-99304-1 (PMC12075579; doi:10.1038/s41598-025-99304-1)

**The synergistic effect of Ginsenoside CK and retinol on UVA-induced photoaging through antioxidant and antiapoptotic mechanisms**

**Supplementary material**

**Figure S1. Effects of ginsenoside CK, retinol (****Vitamin A, VA), hydroxypinacolone retinoate (HPR) and retinol palmitate (VAPA) on cytotoxicity of HaCaT cells determined using MTT assay.** (A) The HaCaT cells were separately treated with CK, Vitamin A, HPR and VAPA at different concentrations cfor 48h. (B-D) HaCaT cells were treated with different concentrations of CK (0.001, 0.005, 0.01, 0.05, 0.1, 0.2 mg/mL) in combination with vitamin A (B) or HPR (C) or VAPA (D) for 48h, respectively.

**Figure S2. Effect of the combination formulation of retinol and CK on MMPs in photoaged cells.** Protein expression levels of MMP2 and MMP3 were determined using Western blot analysis in HaCaT cells treated with CK (0.001, 0.005, 0.01 mg/mL) with or without VA (A), HPR (B) or VAPA (C) for 24 hours. GAPDH was used as a control protein. Quantification charts were listed on the right. **P < 0.01 vs UVA treatment group. ^#^P < 0.05, ^##^P < 0.01 vs VA/HPR/VAPA alone treatment group.

**Figure S1**


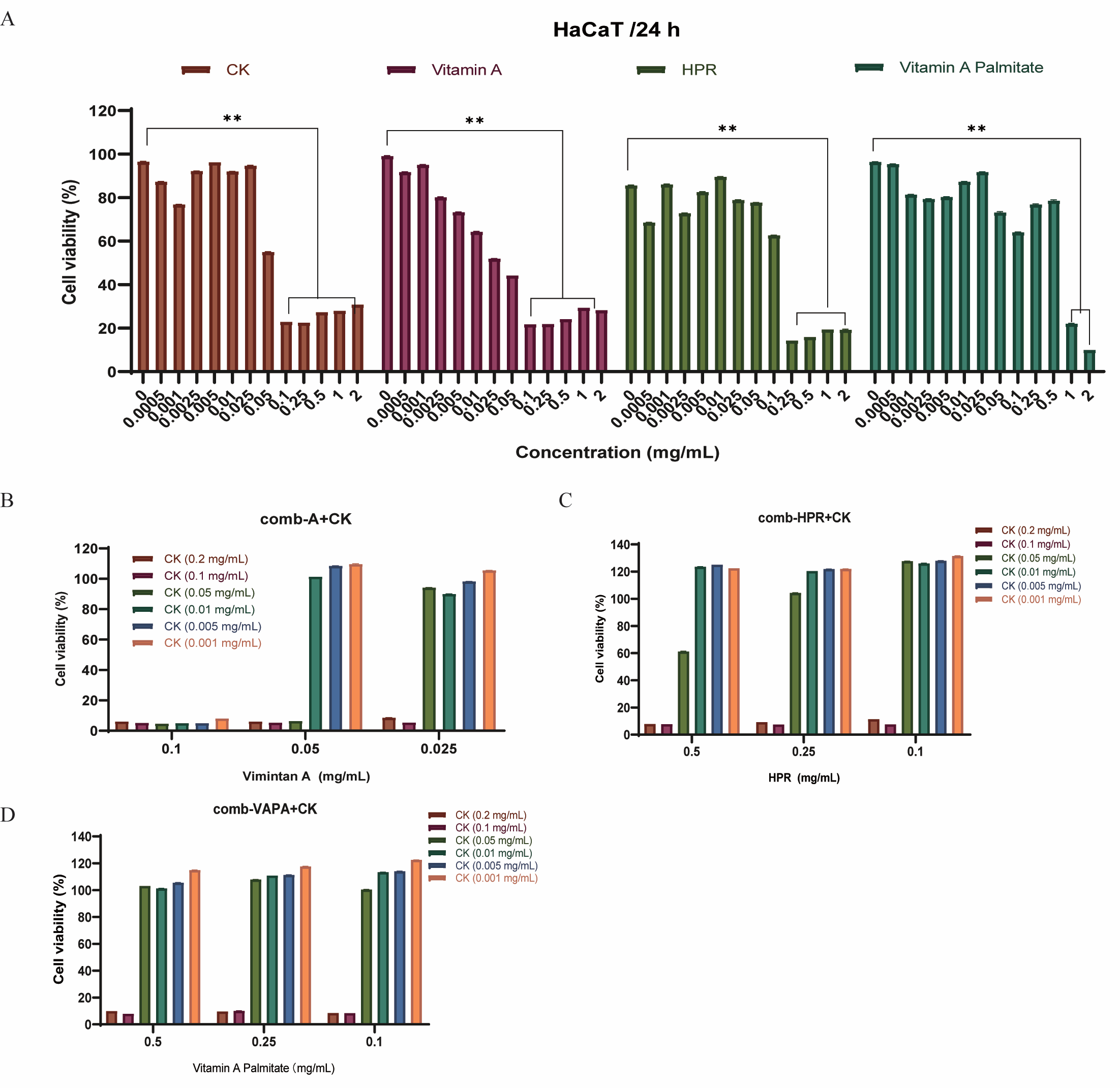


**Figure S2**


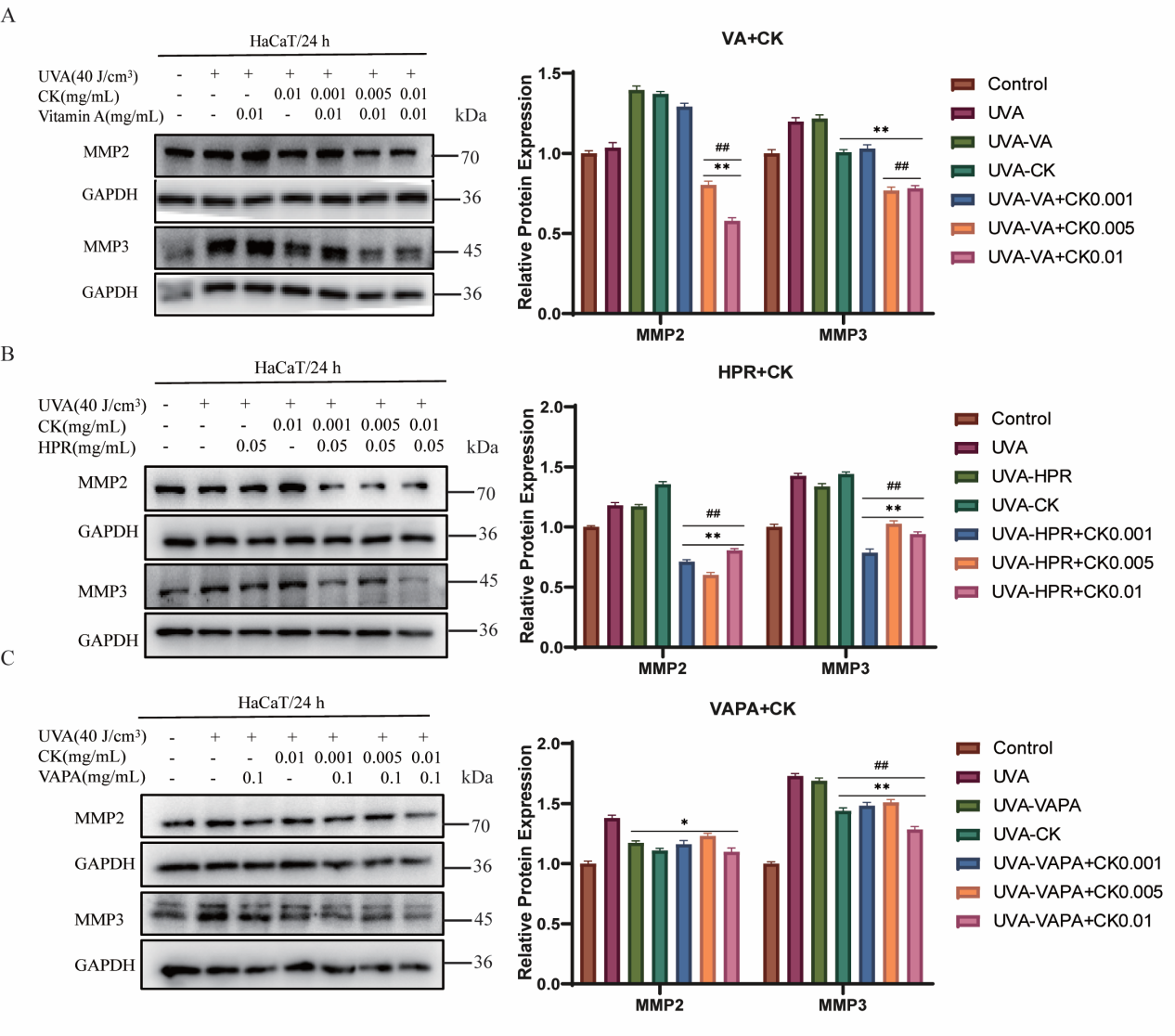

Supplement: Supplementary file 1 — Supplementary Material 1 [file 41598_2025_99304_MOESM1_ESM.docx]
