# Supplementary material for: Ginsenoside CK and retinol on UVA-induced photoaging exert the synergistic effect through antioxidant and antiapoptotic mechanisms
Source: Sci Rep. 2025 May 13;15:16664. doi: 10.1038/s41598-025-99304-1 (PMC12075579; doi:10.1038/s41598-025-99304-1)

Figure 3A

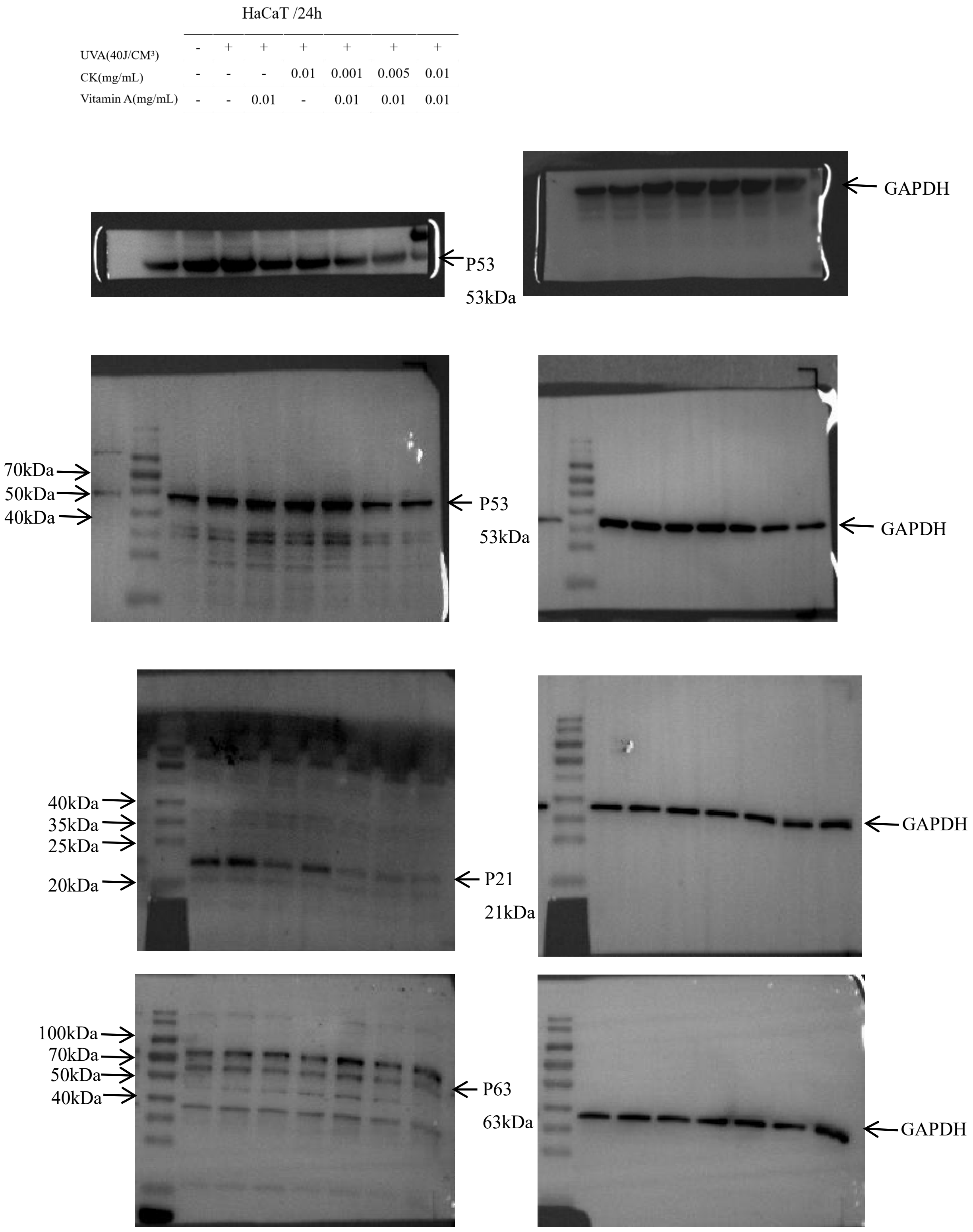
HaCaT /24h
UVA(40J/CM³) - + + + + + +
CK(mg/mL) - - - 0.01 0.001 0.005 0.01
Vitamin A(mg/mL) - - 0.01 - 0.01 0.01 0.01
P53
53kDa
GAPDH
70kDa
50kDa
40kDa
P53
53kDa
GAPDH
40kDa
35kDa
25kDa
20kDa
P21
21kDa
GAPDH
100kDa
70kDa
50kDa
40kDa
P63
63kDa
GAPDH

Figure 3B

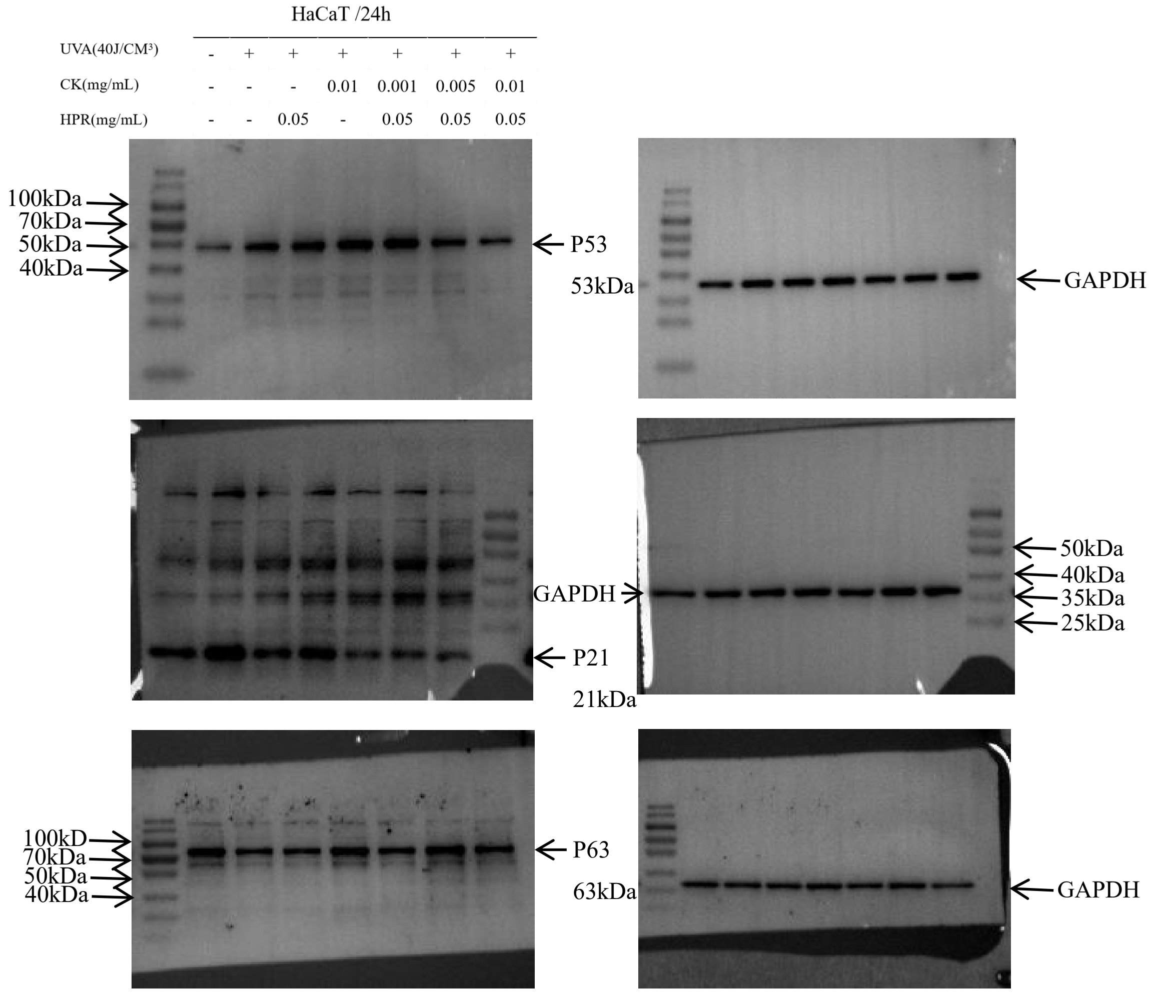
HaCaT /24h
UVA(40J/CM³) - + + + + + +
CK(mg/mL) - - - 0.01 0.001 0.005 0.01
HPR(mg/mL) - - 0.05 - 0.05 0.05 0.05
100kDa
70kDa
50kDa
40kDa
P53
53kDa
GAPDH
GAPDH
P21
21kDa
50kDa
40kDa
35kDa
25kDa
100kD
70kDa
50kDa
40kDa
P63
63kDa
GAPDH

Figure 3C

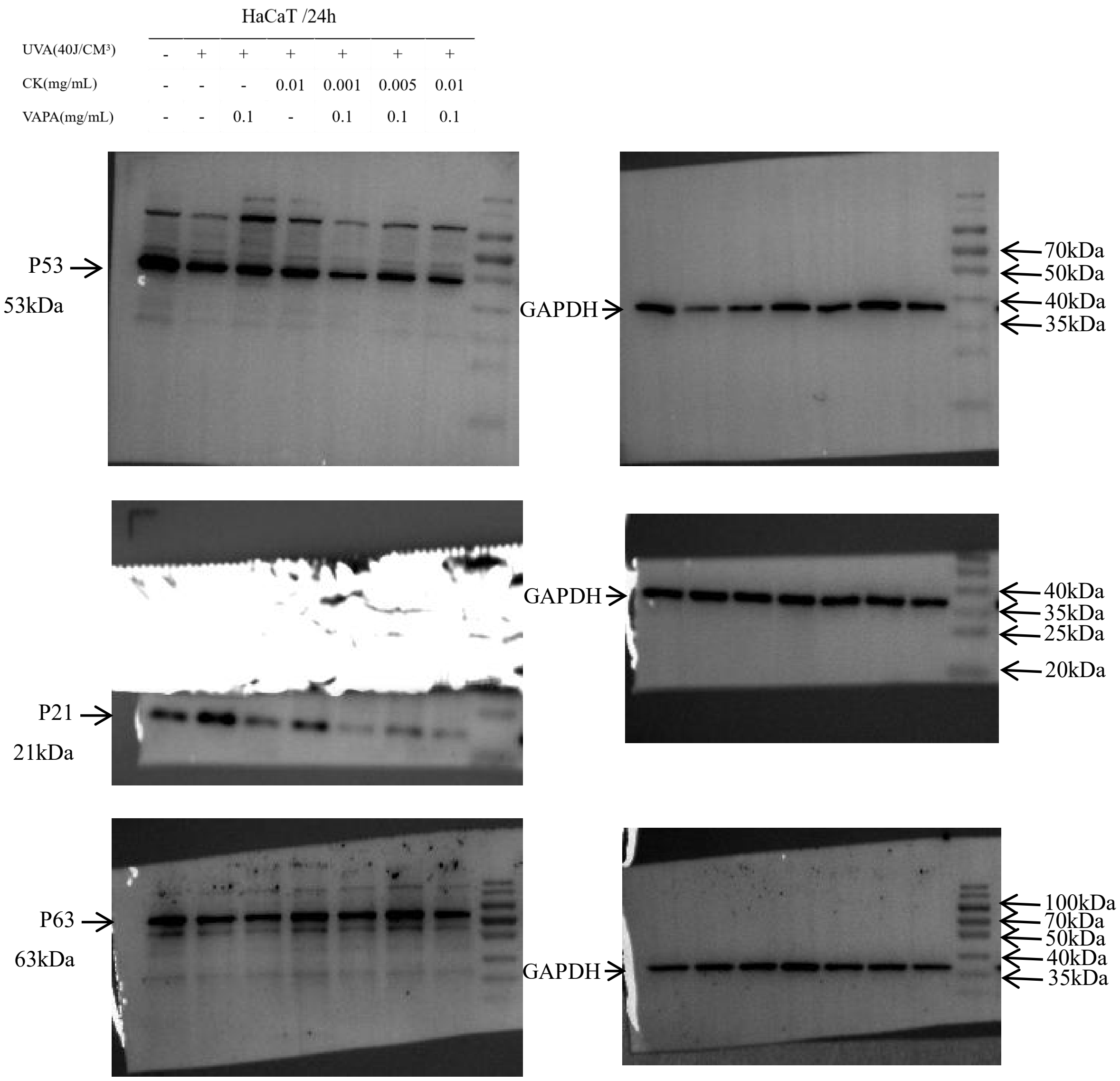
HaCaT /24h
UVA(40J/CM³) - + + + + + +
CK(mg/mL) - - - 0.01 0.001 0.005 0.01
VAPA(mg/mL) - - 0.1 - 0.1 0.1 0.1
P53
53kDa
GAPDH
70kDa
50kDa
40kDa
35kDa
P21
21kDa
GAPDH
40kDa
35kDa
25kDa
20kDa
P63
63kDa
GAPDH
100kDa
70kDa
50kDa
40kDa
35kDa

Figure 4A

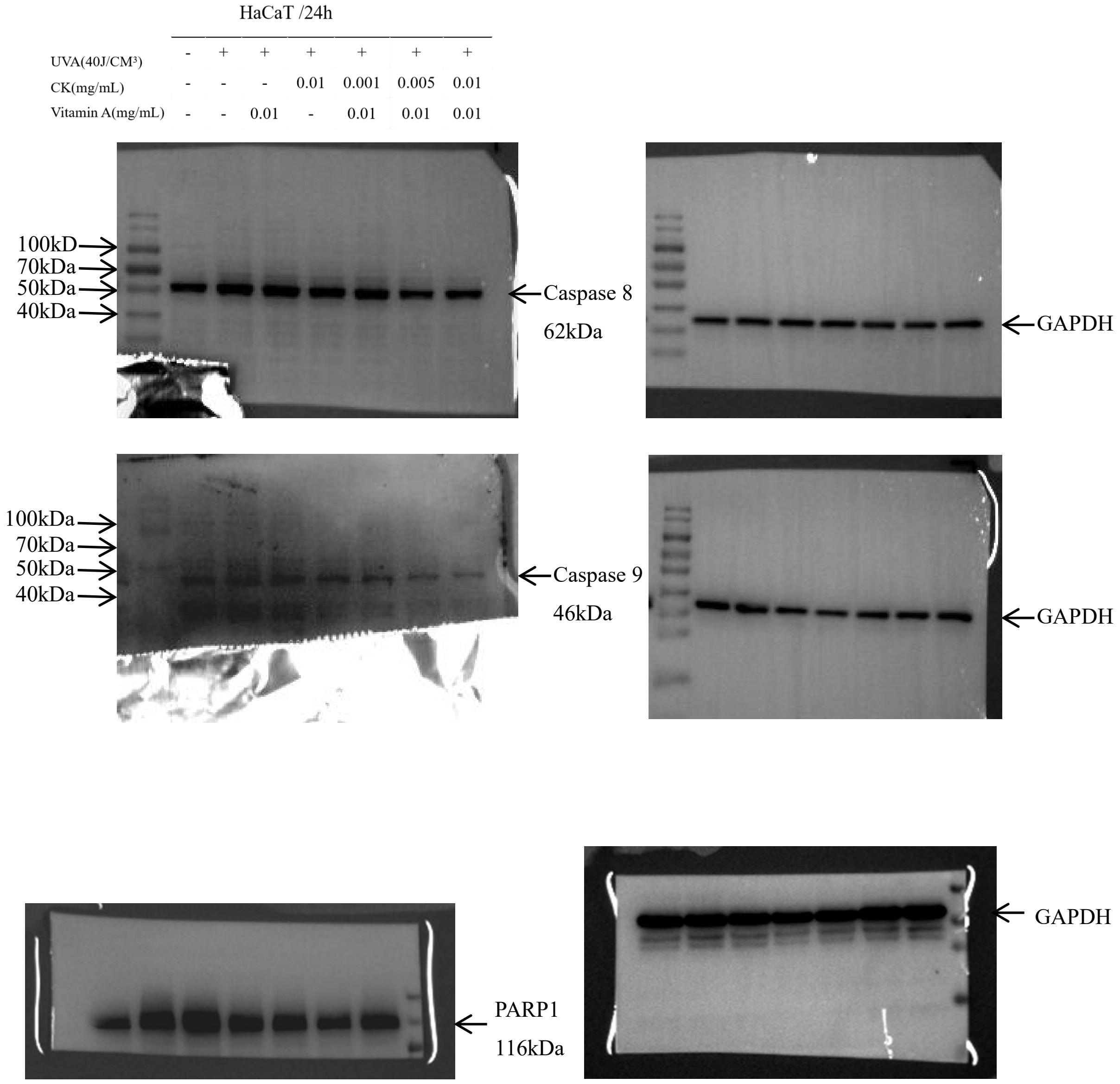

HaCaT /24h
UVA(40J/CM³) - + + + + + +
CK(mg/mL) - - - 0.01 0.001 0.005 0.01
Vitamin A(mg/mL) - - 0.01 - 0.01 0.01 0.01
100kD
70kDa
50kDa
40kDa
Caspase 8
62kDa
GAPDH
100kDa
70kDa
50kDa
40kDa
Caspase 9
46kDa
GAPDH
PARP1
116kDa
GAPDH

Figure 4B

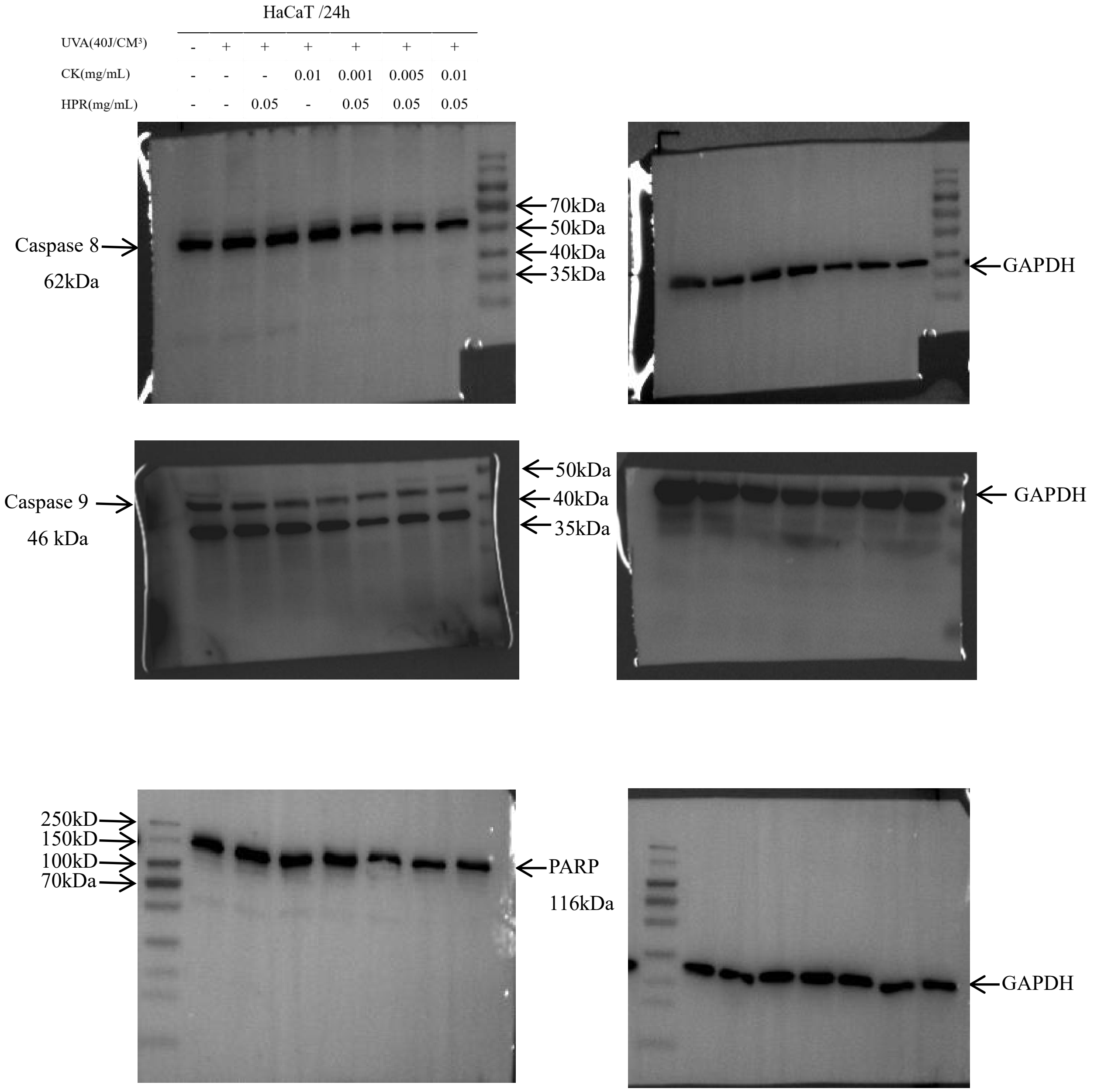
HaCaT /24h
UVA(40J/CM³) - + + + + + +
CK(mg/mL) - - - 0.01 0.001 0.005 0.01
HPR(mg/mL) - - 0.05 - 0.05 0.05 0.05
Caspase 8
62kDa
70kDa
50kDa
40kDa
35kDa
GAPDH
Caspase 9
46 kDa
50kDa
40kDa
35kDa
GAPDH
250kD
150kD
100kD
70kDa
PARP
116kDa
GAPDH

Figure 4C

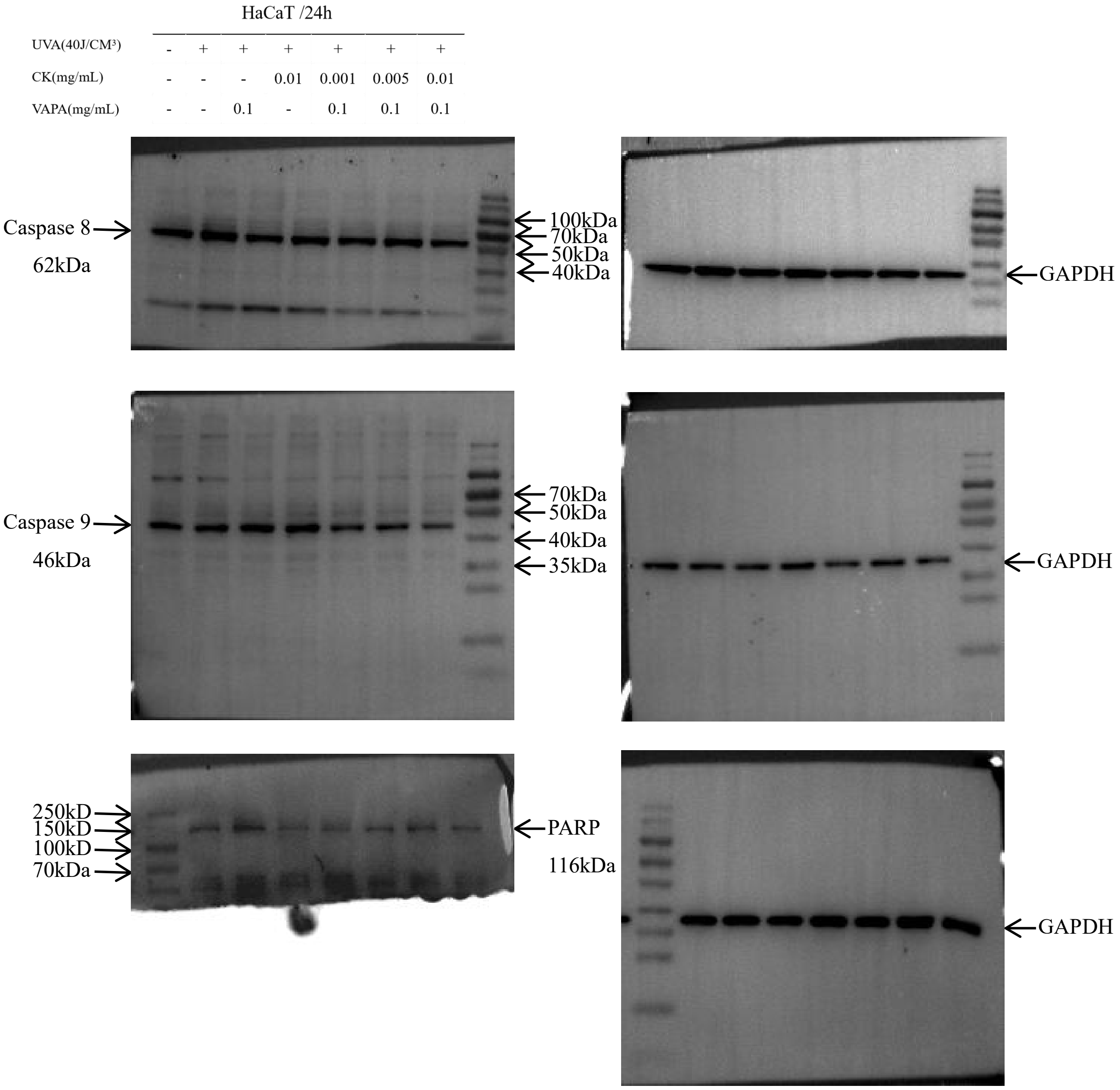
HaCaT /24h
UVA(40J/CM³) - + + + + + +
CK(mg/mL) - - - 0.01 0.001 0.005 0.01
VAPA(mg/mL) - - 0.1 - 0.1 0.1 0.1
Caspase 8
62kDa
100kDa
70kDa
50kDa
40kDa
GAPDH
Caspase 9
46kDa
70kDa
50kDa
40kDa
35kDa
GAPDH
250kD
150kD
100kD
70kDa
PARP
116kDa
GAPDH

Figure 5A

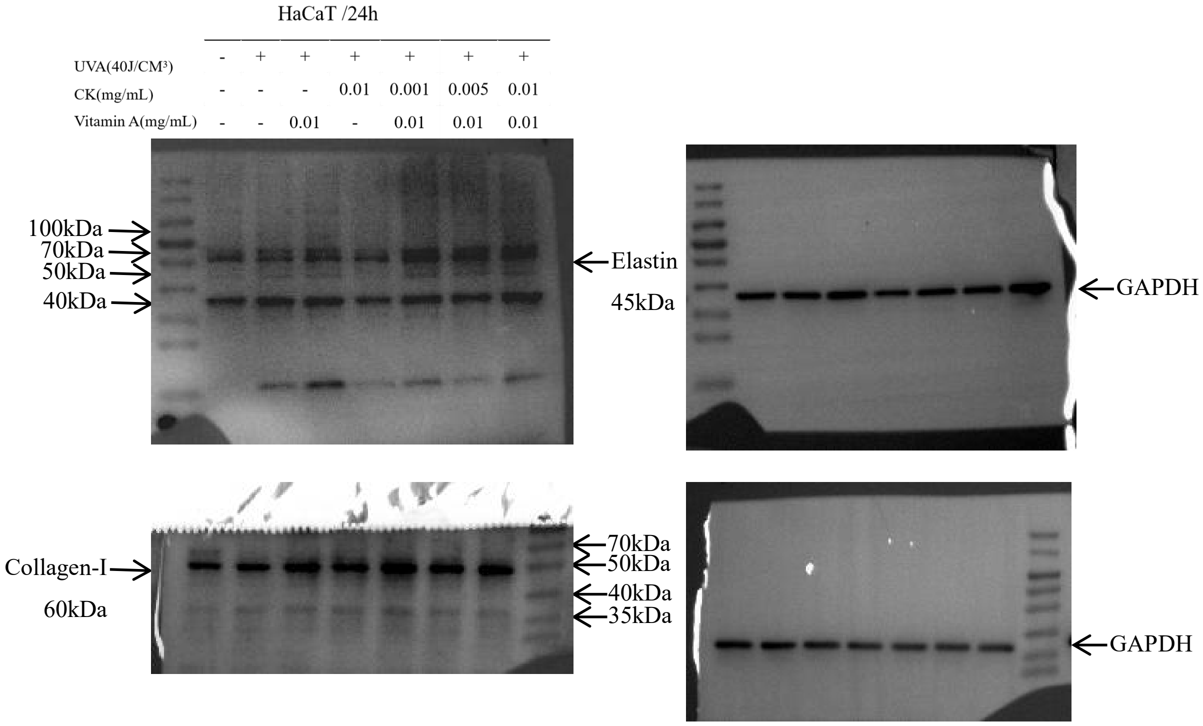
HaCaT /24h
UVA(40J/CM³) - + + + + + +
CK(mg/mL) - - - 0.01 0.001 0.005 0.01
Vitamin A(mg/mL) - - 0.01 - 0.01 0.01 0.01
100kDa
70kDa
50kDa
40kDa
Elastin
45kDa
GAPDH
Collagen-I
60kDa
70kDa
50kDa
40kDa
35kDa
GAPDH

Figure 5B

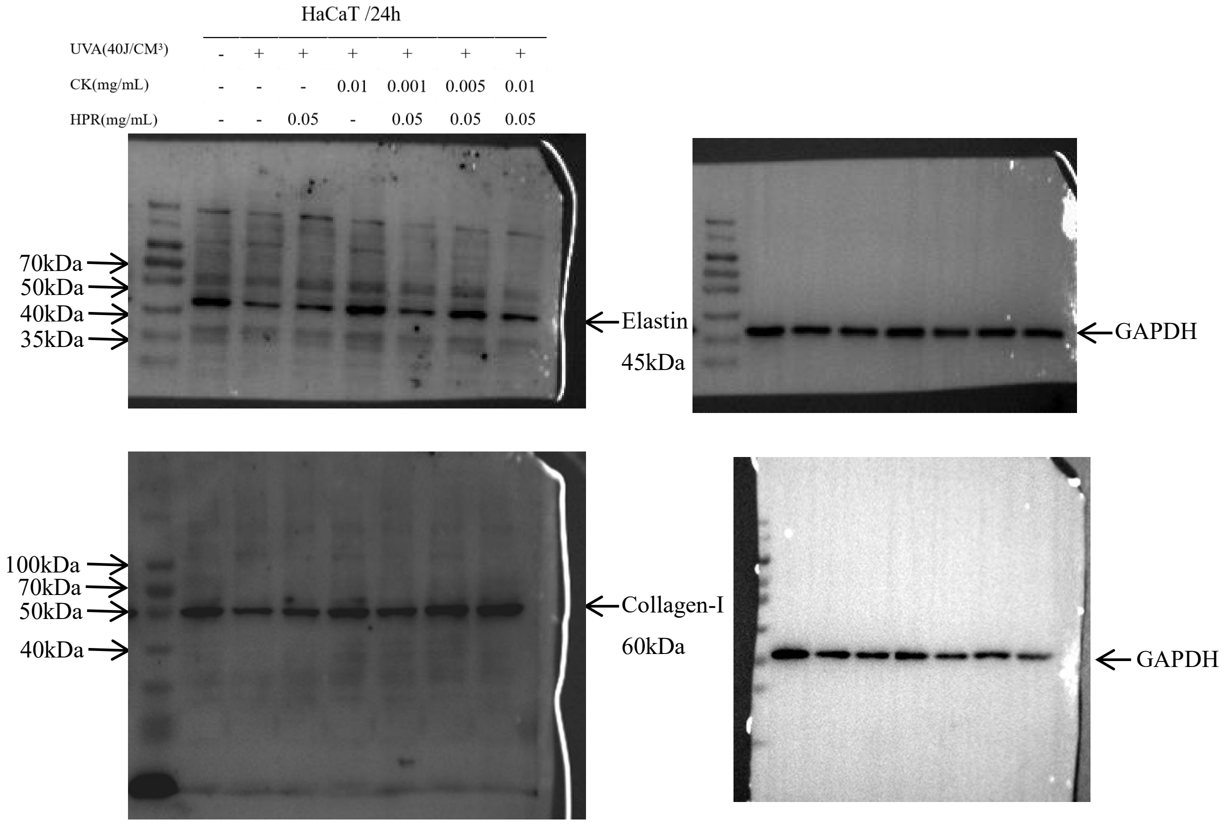
HaCaT /24h
UVA(40J/CM³) - + + + + + +
CK(mg/mL) - - - 0.01 0.001 0.005 0.01
HPR(mg/mL) - - 0.05 - 0.05 0.05 0.05
70kDa
50kDa
40kDa
35kDa
Elastin
45kDa
GAPDH
100kDa
70kDa
50kDa
40kDa
Collagen-I
60kDa
GAPDH

Figure 5C

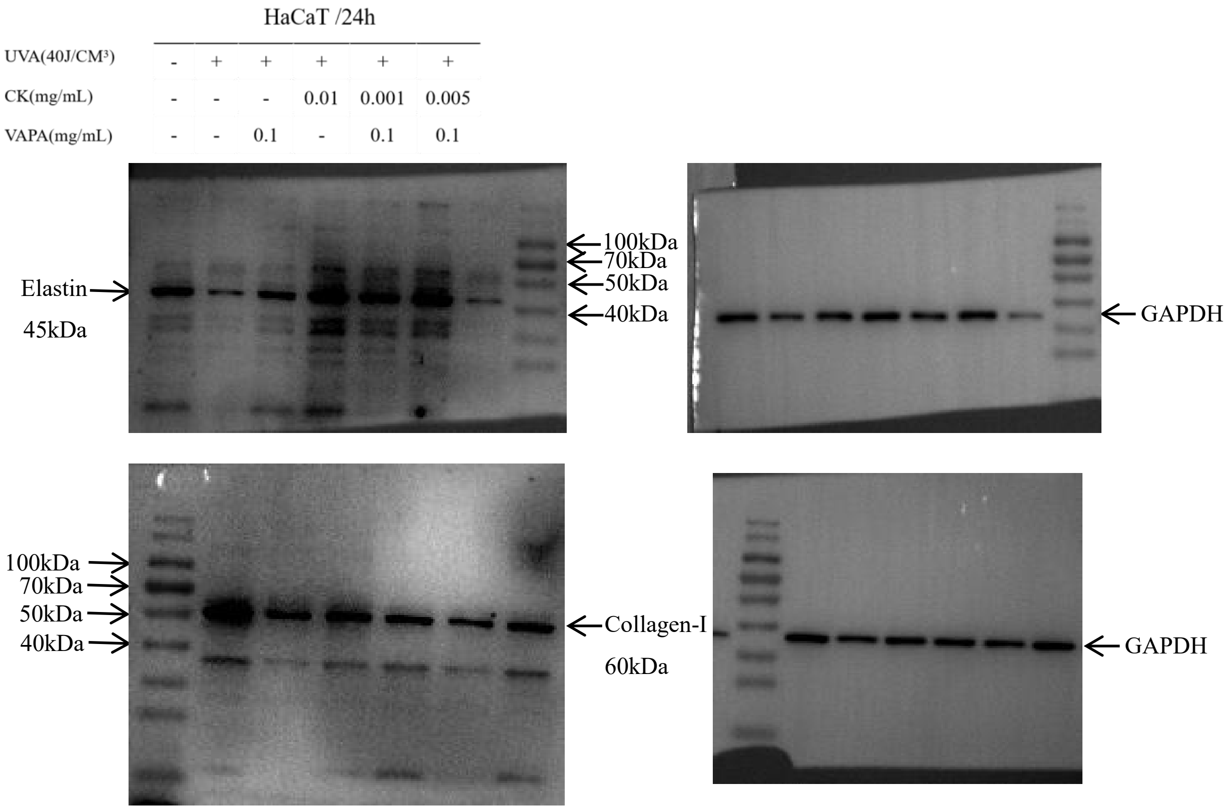
HaCaT /24h
UVA(40J/CM³) - + + + + +
CK(mg/mL) - - - 0.01 0.001 0.005
VAPA(mg/mL) - - 0.1 - 0.1 0.1
Elastin
45kDa
100kDa
70kDa
50kDa
40kDa
GAPDH
100kDa
70kDa
50kDa
40kDa
Collagen-I
60kDa
GAPDH

Supplementary figure 2A

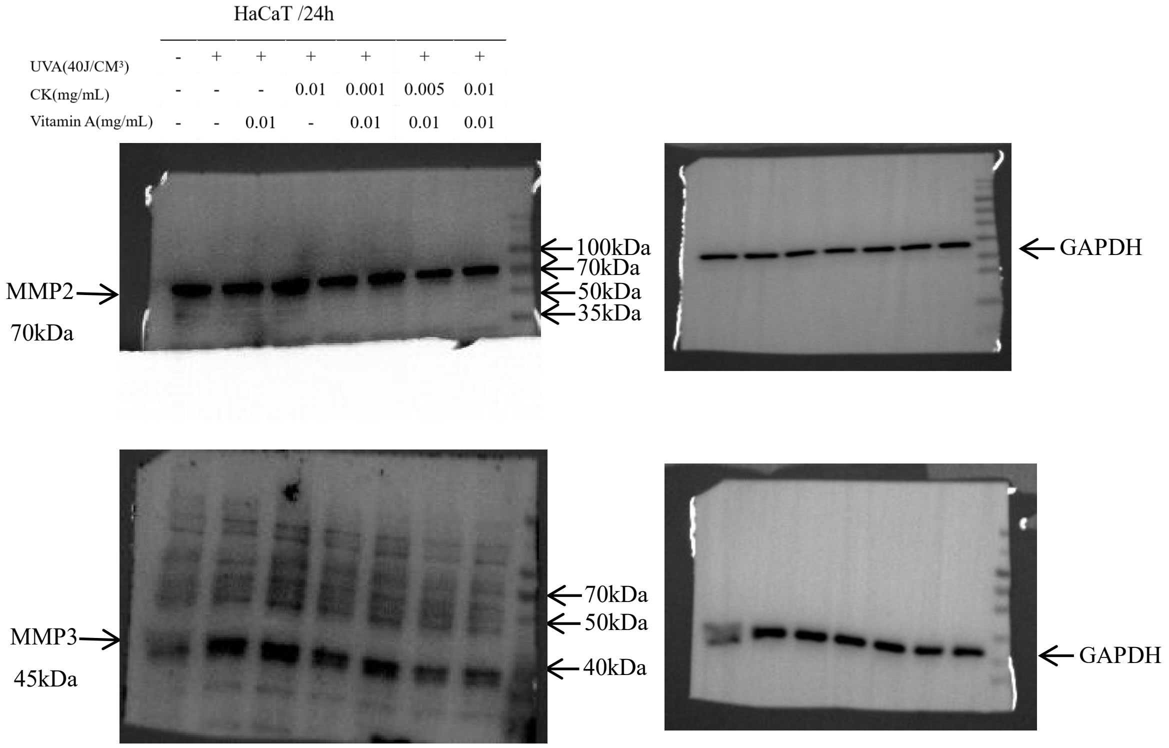

Supplementary figure 2B

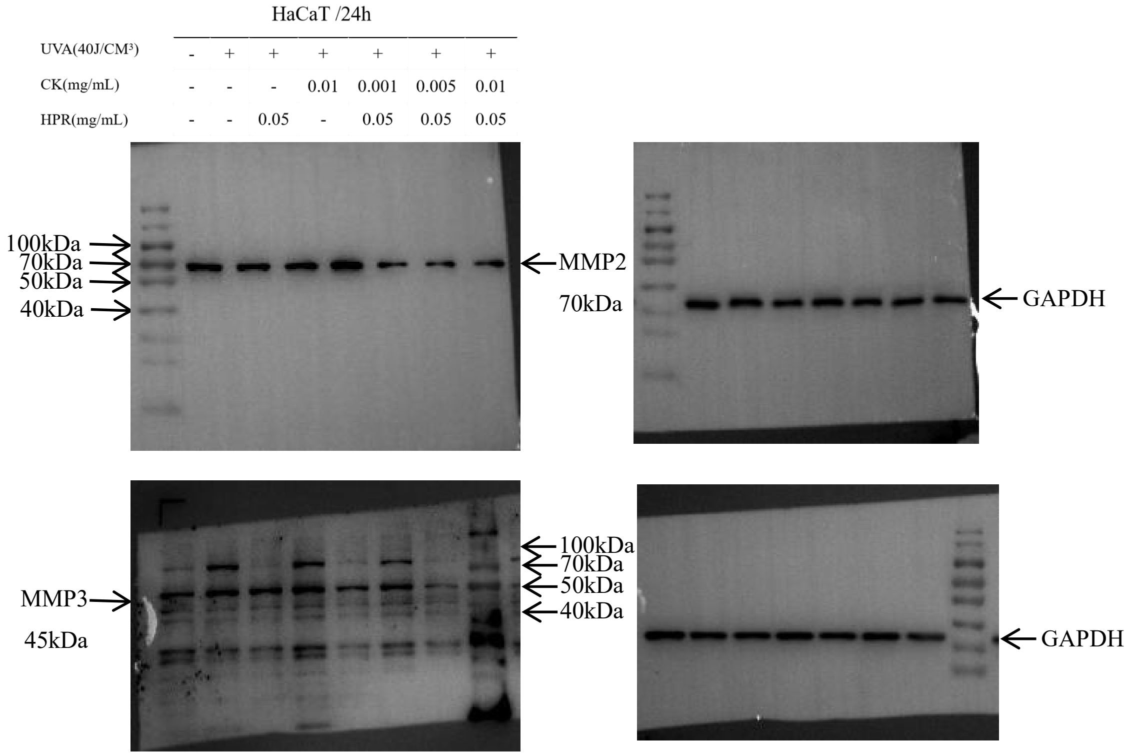

Supplementary figure 2C

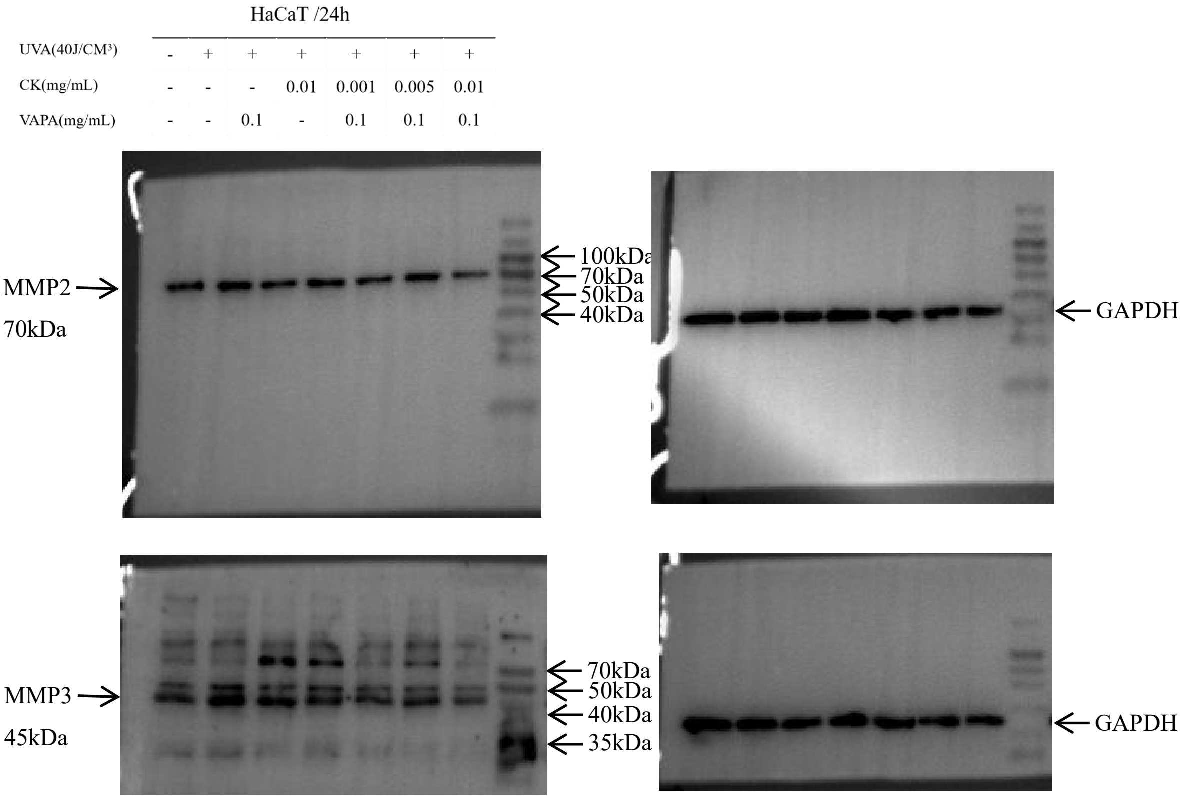

Supplement: Supplementary file 2 — Supplementary Material 2 [file 41598_2025_99304_MOESM2_ESM.pdf]
